# The Role of River Discharge and Geometric Structure on Diurnal Tidal Dynamics, Alabama, USA

**DOI:** 10.1029/2021JC018007

**Published:** 2022-03-28

**Authors:** Steven L. Dykstra, Brian Dzwonkowski, Raymond Torres

**Affiliations:** ^1^ School of Earth, Ocean, and Environment University of South Carolina Columbia SC USA; ^2^ Department of Marine Sciences University of South Alabama Dauphin Island Sea Lab Dauphin Island AL USA; ^3^ Dauphin Island Sea Lab Dauphin Island AL USA

**Keywords:** fluvial‐marine, Mobile Bay, longitudinal, geomorphology, tidal river, estuary

## Abstract

As tides propagate inland, they become distorted by channel geometry and river discharge. Tidal dynamics in fluvial‐marine transitions are commonly observed in high‐energy tidal environments with relatively steady river conditions, leaving the effects of variable river discharge on tides and longitudinal changes poorly understood. To study the effects of variable river discharge on tide‐river interactions, we studied a low‐energy tidal environment where river discharge ranges several orders of magnitude, the diurnal microtidal Tombigbee River‐Mobile Bay fluvial‐marine transition, using water level and velocity observations from 21 stations. Results showed that diurnal tidal attenuation was reduced by the width convergence in seaward reaches and height convergence of the landward backwater reaches, with the channel convergence change location ∼40–50 km inland of the bayhead and seaward of the largest bifurcation. River events amplified tides in seaward regions and attenuated tides in landward regions. This created a region of river‐induced peak amplitude seaward of the flood limit (i.e., bidirectional‐unidirectional current transition), allowing more tidal energy to propagate inland. Tidal currents were attenuated and delayed more by river discharge than water levels, making the phase lag dynamic. The river impacts on the tides were delineated longitudinally and shifted seaward as river discharge increased, ranging up to ∼180 km. Results indicated the longitudinal shifts of river impacts on tides in alluvial systems can be estimated analytically using the ratio of river discharge to tidal discharge and the geometric convergence of the system. Our simple analytical theory provides a pathway for understanding the tide‐river‐geomorphic equilibrium along increasingly dynamic coasts.

## Key Points

- Along the fluvial‐marine transition, river discharge attenuated tides in landward regions and amplified tides in seaward regions
- Regions of bidirectional tidal flow became unidirectional as river discharge increased, shifting the tidal river ∼180 km seaward
- Planform channel geometry (e.g., width convergence) may not be a robust indicator of the transition in tide‐river hydrodynamics

## 1 Introduction

Most ports and mega cities are located where rivers reach the sea, concentrating the human population, economies, and infrastructure in regions where water levels and currents are controlled by rivers and tides. These fluvial‐marine transitions extend far inland to the head of tides O(10‐1,000 km), connecting the land to the sea and forming critical links for biogeochemical cycles, source to sink sediment dynamics, and ecosystems. Tide and river dynamics are described analytically based on their geometries and are highly variable, changing on timescales of hours to days. When tides and rivers interact, the processes are strongly nonlinear and shape the local channel or estuary geometry (Dalrymple & Choi, 2007; Haigh et al., 2020; Sulaiman et al., 2021). To investigate tide‐river‐geomorphic interactions, we focus on the longitudinal variability of the regions where flood‐tide flows cease to exist and where tides become entirely damped, comparing our results from a diurnal tidal system with existing predictors to analytical theory.

Tides are generated by astronomical forces and propagate as long waves through the ocean. Tidal waves are orbital, like wind waves, with a vertical component observed in water level and a horizontal component observed in currents (herein vertical wave/tide and horizontal wave/tide, respectively). Tides can amplify or attenuate along coasts, especially after entering confined bays and rivers as forced waves where the forced tidal waves become further modulated by landward decreasing cross‐sectional areas, bed friction, and river flow (Friedrichs, 2010). Therefore, modulation can make tidal currents more effective at scouring and transporting sediment, shaping their geomorphic environments, as well as mixing surface and bottom waters, which can reduce salt intrusion, oxygenate bottom waters, and bring nutrients to the surface (Dalrymple & Choi, 2007; Ralston & Geyer, 2019). While semidiurnal tides often have larger amplitudes, some of the longest historical records and climate models show diurnal tides are amplifying faster and have experienced larger phase changes (Cartwright, 1971; Green, 2010). If these trends continue, some regions will become more diurnal and similar to the modern diurnal areas of the western and south Pacific Ocean, the west coast of Australia, and the Gulf of Mexico/Caribbean Sea.

**Figure 1:**
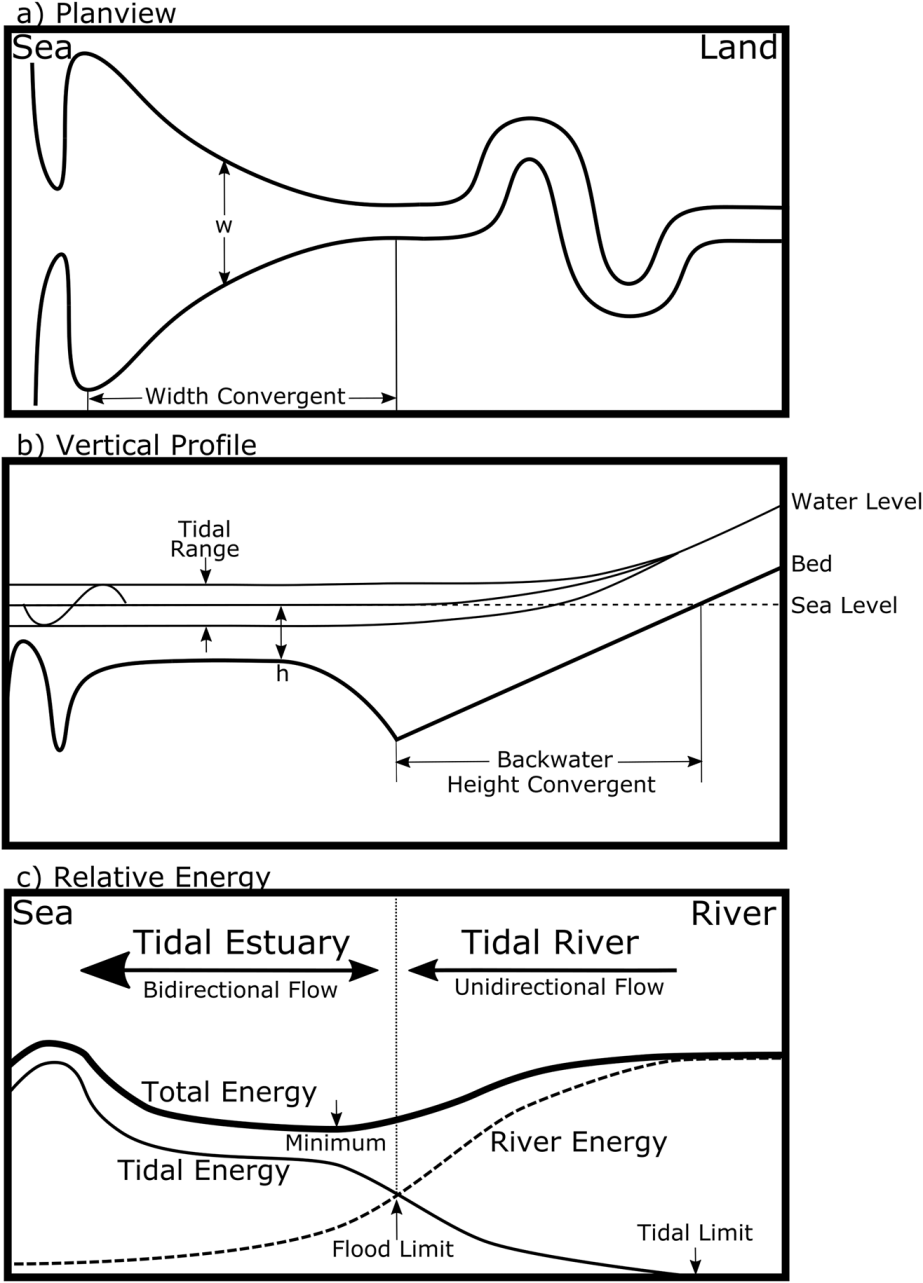
Cartoon of a fluvial‐marine transition, showing the (a) planview, (b) vertical profile, and (c) relative river and tidal energy. (Modified from Dalrymple & Choi, 2007).

The transition from tidal to river dominant flow creates a large change in the equilibrium geometry of fluvial‐marine transitions (Kästner et al., 2017; Sassi et al., 2012; Wright et al., 1973). Tidally dominated areas are similar to the sea, commonly wide and flat. For tidal dominated areas in alluvial systems at geomorphic equilibrium, the width converges landward forming a trumpet shape and the shallowest region is a bar at the mouth (Figure 1a; Dronkers, 2017; Friedrichs & Aubrey, 1994; Kästner et al., 2017; Valle‐Levinson et al., 2019). These reaches can be open bays, divided into multiple deltaic distributaries, or a combination of both (e.g., bayhead delta; Dalrymple & Choi, 2007). River‐dominated regions are similar to low lying fluvial environments, relatively narrow and sinuous. At equilibrium, the depth is variable like inland reaches, but increases seaward, forming a backwater environment where the riverbed drops below sea level (Figure 1b). The diverging bed and water surface decrease river flow and sediment transport, increasing deposition and river sinuosity (Ganti et al., 2019; Lane, 1957; Lazarus & Constantine, 2013; Leuven et al., 2021; Myrick & Leopold, 1963). It has been reported that where the tide and river‐dominated reaches meet is the deepest region of the fluvial‐marine transition and has little change in cross‐sectional area (Gugliotta & Saito, 2019; Kästner et al., 2017).

Fluvial‐marine transitions have flow contributions from each end with peak tidal currents decaying in a landward direction and river currents decaying in a seaward direction. The balance point occurs where flood tides are no longer able to reverse river flow and is herein called the flood limit (Figure 1c). The flood limit commonly delineates analytical solutions into the bidirectional flowing tidal estuary (herein estuary) and the tidal river, where unidirectional flow is tidally modulated (e.g., Cai et al., 2014; Friedrichs, 2010; Jay & Flinchem, 1997; van Rijn, 2011). The landward extent of the tidal river is the tidal limit, where the incident tidal wave at the mouth has fully decayed (Figure 1c). Tide‐river interactions may change with tidal variability, which can range several fold over spring‐neap cycles, but river discharge often has a larger range, up to several orders of magnitude, making tide‐river interactions strongly dependent on river discharge.

Observations show inconsistent tidal responses to river events, amplifying tides in some locations and attenuating them in others. Studies capturing most of the spatial extent of a fluvial‐marine transition, including those on St. Lawrence (Godin, 1985, 1999; Matte et al., 2014), Mekong (McLachlan et al., 2017), Yangtze (Guo et al., 2015), and Guadalquivir River (Losada et al., 2017), generally show that terrestrial flood events amplify vertical tides in seaward reaches and attenuate vertical tides in landward reaches, but do not delineate where this change occurs. While the mechanism causing these reach‐specific tidal responses may not be identified, other studies indicate that river‐associated tidal amplification is attributed to reduced friction. The reduced friction is caused by high stratification from large fluxes of freshwater, lowering the density of the surface layer, or more sediments, increasing the water density near the bed (Díez‐Minguito, Baquerizo, Ortega‐Sanchez, Navarro, et al., 2012; Talke et al., 2009). River‐associated tidal attenuation is attributed to high friction from the river flow and friction from river‐tide interactions (Buschman et al., 2009; Godin, 1985; Kästner et al., 2019).

### 1.1 Tide‐River‐Channel Interactions

River processes interact with the geomorphology and are self‐organizing, reshaping their geometries toward equilibrium (e.g., Leopold et al., 1964; Phillips & Jerolmack, 2016), similar to tide‐geomorphic interactions (Dronkers, 2017). For example, in alluvial estuaries, the tidal energy loss from the friction is balanced by width convergence (e.g., Figure 1a), causing partial reflection of the tidal wave. When the tidal amplitude is constant across the reach, it is called an *ideal* estuary. If friction dominates, the estuary is hyposynchronous and causes the wave to attenuate, the phase lag to decrease (i.e., the phase difference between horizontal and vertical tidal components), and the phase and wavenumber to increase. If convergence dominates, the estuary is hypersynchronous and causes the wave to amplify, the phase lag to increase, and the phase and wavenumber to decrease (Friedrichs, 2010; Friedrichs & Aubrey, 1994). Estuarine convergence in new empirical relationships is shown to decrease for systems with higher river discharge, due in part to the larger river width, indicating that estuarine morphology and tides reach an equilibrium that may change with river discharge (Dronkers, 2017; Leuven et al., 2018).

Tide and river processes are also interpreted from geologic records from depositional patterns, bedforms/structure, and trace fossil assemblages (Davis, 2011; Sisulak & Dashtgard, 2012; van den Berg & Boersma, 2007). Stratigraphic reconstructions (e.g., facies) capture the longitudinal change of fluvial‐marine transitions, but some regions have alternating patterns that suggest reaches cyclically switch between river and tidal dominant environments (Davis, 2011; Rossi et al., 2017). Dalrymple et al. (2015) hypothesize the alternating patterns are caused by tide‐river processes shifting seaward and landward with the rise and fall of river discharge. However, longitudinal shifts are poorly documented in modern equivalent systems, leaving the hypothesis unsubstantiated (Dalrymple et al., 2015).

### 1.2 Motivation for New Observations

Longitudinal changes in tidal dynamics are not commonly translated from observations of river induced tidal attenuation and may be a result of small changes due to previous literature focusing on similar systems (i.e., macrotidal/mesotidal, semidiurnal) with small ranges in river discharge (e.g., an order of magnitude or less). The ratio of river discharge to tidal discharge (*Q*_rf_ = Q_r_/Q_t_) is relatively stable, making tide‐river interactions and their geomorphic effects also stable (Zhou et al., 2014). Thus, studies on river induced tidal attenuation often ignore the effects of discharge changes (e.g., Horrevoets et al., 2004). In contrast, recent observations of the microtidal Kapuas River (tidal range 0.35–1.35 m) show an order‐of‐magnitude increase in discharge causes strong tidal attenuation and shifts the tidal limit over 130 km seaward (Kästner et al., 2017). The tidal period also strongly affects wave energy. The dominant tidal specie (e.g., diurnal [D1], semidiurnal [D2]) strongly affects the tidal friction of all species, making D1 tidal attenuation in semidiurnal environments dependent on D2 tides and not D1 tides (Godin, 1985; Jay & Flinchem, 1997). For simplicity, the tidal theory is often derived explicitly for D2 waves (e.g., van Rijn, 2011; Winterwerp & Wang, 2013), limiting applications to diurnal environments and may partially explain why there are no studies of tide‐river interactions in diurnal systems.

An advanced understanding of tidal and river hydrodynamics is also limited by studies that principally rely on water level observations (e.g., Jay et al., 2015; Webb & Marr, 2016). This is problematic because the water level only captures the vertical component of the tidal wave. For discharge/currents, friction, and transport, the horizontal wave component, captured with velocity observations, is needed. Horizontal waves can be theoretically estimated with water levels, although this estimation assumes that the vertical and horizontal waves are congruent and temporally offset with a constant phase lag (Buschman et al., 2009; Sassi & Hoitink, 2013), which may not be appropriate for many realistic environments (Friedrichs, 2010). In width‐converging environments, the convergence rate is positively related to the vertical amplitude and inversely related to the horizontal amplitudes (Friedrichs, 2010). For height‐convergent environments, new theory shows that river discharge attenuates horizontal waves faster than vertical waves (Kästner et al., 2019). Direct observations of horizontal and vertical waves in the Guadalquivir River show river events can strongly affect the phase lag between horizontal and vertical waves (Díez‐Minguito, Baquerizo, Ortega‐Sanchez, Navarro, & Losada, 2012; Losada et al., 2017). This notwithstanding, very few fluvial‐marine transitions have long‐term velocity observations at multiple locations, leaving tidal theory related to horizontal wave propagation largely untested.

The purpose of this study is to quantitively assess tidal attenuation dynamics across the fluvial‐marine transition. Thus, we hypothesize that longitudinal shifts in regions dominated by tides or rivers along a fluvial‐marine transition are caused by variable river discharge (i.e., Dalrymple et al., 2015). Our objectives are to (a) observe river discharge and geometry effects on the longitudinal variability of tides (vertical and horizontal components) from the width convergent region of an estuary through a height convergent backwater, (b) analytically capture longitudinal shifts of tide‐river interactions, and (c) identify differences in how diurnal tides may affect hydrodynamics and geomorphology when compared to semidiurnal tides. The objectives are completed using long‐term water level and velocity observations, spatial data (e.g., LiDAR), and analytical equations to capture the dynamics of a diurnal microtidal environment, the Mobile Bay‐Tombigbee River fluvial‐marine transition in Alabama, USA.

## 2 Theory

Tidal waves propagating along a fluvial‐marine transition are primarily modulated by friction, basin shape, and subtidal flows, which generally arise from geomorphic and river discharge interactions. The primary geomorphic effects on tides are bed stress, reflectance (e.g., convergence, dam), and resonance (Friedrichs, 2010; Jay, 1991). The primary river effects on tides are through river currents (e.g., friction), the discharge volume (e.g., reduces tidal prism), and density (e.g., stratification; Cai et al., 2014).

### 2.1 Tidal Controls on the Dimensions of *Ideal* Estuaries

For tide‐geomorphic interactions, convergence is found using an e‐folding length based on the reach width (w) or height (h):

(1a) $$w=w_{0}e^{-x/L_{w}}$$

(1b) $$h=h_{0}e^{-x/L_{h}}$$

where L_w_ is the width e‐folding length, L_h_ is the height e‐folding length, and the subscript of 0 indicates the longitudinal location inland where landward convergence begins. This location may be offset from the estuary mouth (e.g., bar‐built estuary; Dronkers, 2017). As the e‐folding length increases, convergence decreases. The cross‐sectional area (*a*) e‐folding length can be solved following van Rijn (2011):

(2) $$L_{a}^{-1}\approx L_{w}^{-1}+L_{h}^{-1}$$

The e‐folding length is used to quantify the role of convergence on tidal amplitude following Talke and Jay (2020):

(3a) $$\eta \left(x\right)\approx \eta_{0}e^{\mu x}$$

where x is the along channel distance inland,

(3b) $$\mu \approx \frac{1}{2L_{a}}-\omega \sqrt{c_{d}\eta_{0}L_{p}}/\sqrt{gh^{3}}$$

Here, μ is the damping rate, ω is the angular frequency (i.e., *ω* = 2π/T, T is tidal period), c_d_ is the drag coefficient, η is the vertical tidal amplitude, L_p_ is the length scale controlling the tidal prism (i.e., distance to the flood limit), and g is gravity. The first term in the damping rate (Equation 3b) is convergence and the second is the damping modulus. Equation (3a), (3b) indicates the vertical tidal amplitude is a balance between convergence, amplifying the vertical tide, and friction, attenuating the tide.

In shallow convergent estuaries where L_w_ << k^−1^, the vertical and horizontal tidal components are closely related through (Friedrichs, 2010):

(4) $$U_{t}=\frac{\eta \omega L_{w}}{h}\frac{w_{b}}{w}$$

where w_b_/w is the mean width (w_b_) over a tidal cycle relative to the channel width (w). Equation 4 indicates tidal velocity, as a measurement of the horizontal tidal wave, is proportional to the vertical tidal amplitude, inversely related to convergence (i.e., convergence is 1/L_w_), and is half the magnitude for diurnal tides, relative to semidiurnal tides. The inverse relationship occurs because convergence partially reflects the tidal wave, which, for the vertical tide that propagates as a transverse wave is in phase and adds to the amplitude, but the horizontal wave, which propagates as a longitudinal wave, is out of phase and subtracts from the amplitude. Thus, tidal modulation may produce different results for the vertical and horizontal components.

When the estuary attains *ideal* geographic attributes at morphodynamic equilibrium and the tidal magnitude is constant, *μ* = 0. Equation 3b can then be rewritten to find the morphodynamic equilibrium of an estuary based on the incident tidal momentum:

(5) $$\frac{\eta_{0}}{T^{2}}\approx \frac{h^{3}g}{16\pi L_{a}^{2}L_{p}c_{d}}$$

where the tidal properties are on the left side and the morphodynamics are on the right. The tidal period and magnitude are inversely related because longer periods have less energy. The tidal momentum scales positively with height and inversely with the length terms and drag coefficient. Because period is squared and magnitude is not, Equation 5 shows that morphodynamics is more sensitive to changes in period and that for a diurnal and a semidiurnal system of similar tidal magnitude, the diurnal system is longer, reflecting the observations of diurnal tides extending further inland than semidiurnal tides (e.g., Godin, 1999; Jay et al., 2015).

The landward river‐dominated reach of fluvial‐marine transitions are primarily affected by marine processes through sea level creating a backwater environment. Here, river events propagate seaward as river flood waves make flow unsteady. The momentum is diffusive (i.e., pressure gradient‐friction balance) and becomes dynamic (i.e., pressure gradient‐friction‐acceleration balance) in tidal dominated reaches (Dykstra & Dzwonkowski, 2020a). The river waves occur later in a seaward direction, like tides in a landward direction, making tide‐river interactions along a longitudinal transect nonconcurrent. Assuming steady river discharge, Kästner et al. (2019) derive the first analytical solutions for tidal waves in backwater reaches, showing tidal waves are modulated by strong river currents and by river water level slope, making height convergence dynamic. While analytical solutions for tidal wave‐river wave interactions are still needed, our study provides an observational exploration of these processes.

### 2.2 The Longitudinal Variability of Tide‐River Interactions

To delineate the tidal and river dominant reaches, Hoitink and Jay (2016) propose a novel method based on the temporal variability of water levels utilizing the nonlinearities of tide‐river interactions. As tides are modulated by river discharge, they become asymmetric and transport water landward. Larger tidal amplitudes transport more water (e.g., Stokes transport). Due to spring‐neap cycles, water is stored in inland reaches, creating a subtidal setup with a fortnight period (e.g., Msf). Where the subtidal variability exceeds tidal variability, the lowest low water occurs during neap tides. With this method, lowest spring tide water levels relative to the lowest neap water levels are lower in the estuary and higher in the tidal river (Hoitink & Jay, 2016; Jay et al., 2015).

Instead of delineating with water level variability, the estuary‐tidal river boundary can be delineated with flow direction as bidirectional or unidirectional at the flood limit. Due to the absence of salt at the flood limit, vertical density gradients and tidal phase have been reported as nearly uniform across the water column of a coastal plain river (Yankovsky et al., 2012), suggesting velocity measured at one location can be used to estimate the cross‐sectionally averaged velocity and discharge. River discharge and peak tidal discharge (Q_t_ = U_t_hw, where U_t_ is the cross‐sectionally averaged peak tidal velocity) can then be used with mass conservation to simulate the longitudinal variability of the flood limit in an *ideal* estuary (i.e., constant tidal amplitude) when convergence is known (Equation (1a), (1b)):

(6a) $$x_{rf}=-L_{w}\;\log\left(\frac{Q_{r}}{w_{0}hU_{t}Q_{rf}}\right)$$

where x_rf_ is the longitudinal location of a given river discharge fraction (Q_rf_ = Q_r_/Q_t_) such as the flood limit (*Q*_rf_ = 1) or location where river flow begins to have negligible tidal damping effects (*Q*_rf_ = 4/3*π* ≈ 0.42; Kästner et al., 2019). Equation 6a can be simplified for the area using Equation 2:

(6b) $$x_{rf}=-L_{a}\;\log\left(\frac{Q_{r}}{a_{0}U_{t}Q_{rf}}\right)$$

Or, if U_t_ is unknown and intertidal effects are negligible (i.e., w_b_/w ≈ 1), x_rf_ can be estimated using water level by substituting Equation 4:

(6c) $$x_{fl}=-L_{w}\;\log\left(\frac{Q_{r}}{w_{0}\eta \omega L_{w}Q_{rf}}\right)$$

Equation 6c shows the longitudinal location of the flood limit has a geomorphic control, largely set by the mouth width and convergence, and can dynamically change with river discharge and tidal amplitude. The log relationship suggests as river discharge increases by one order of magnitude the tide‐river interactions will shift two e‐folding lengths seaward.

## 3 Site Description: Tombigbee River‐Mobile Bay Fluvial‐Marine Transition

**Figure 2:**
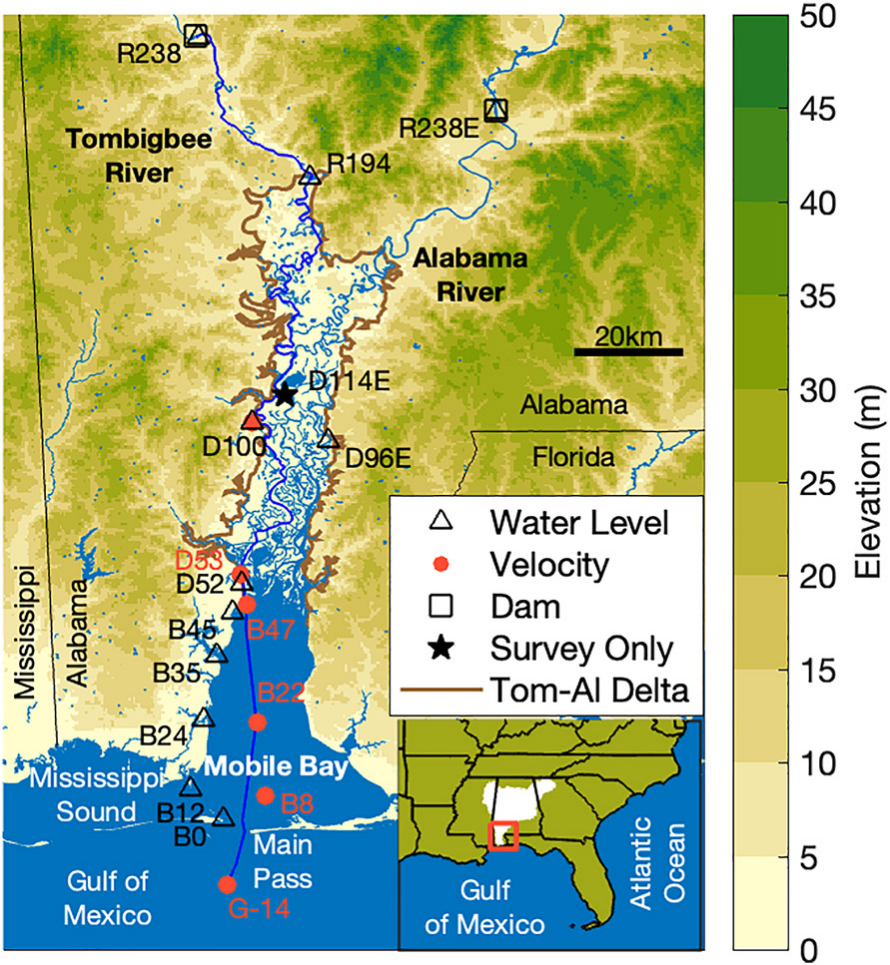
A map of the coastal region of Alabama showing the location of the long‐term stations. The longitudinal transect along the Gulf of Mexico, Mobile Bay, Mobile Distributary, and Tombigbee River is shown with a dark blue line.

The Tombigbee River‐Mobile Bay system in coastal Alabama provided a unique opportunity for this study with a high concentration of long‐term datasets in a representative system (Figure 2, Table S1 in Supporting Information S1). The flat shallow bay is a bar‐built drowned valley estuary, 48 km long, averages 3.5 m deep (NOAA National Geophysical Data Center, 2009), and is highly stratified throughout much of the year (Schroeder et al., 1990). River discharge entering Mobile Bay comes primarily from the Tombigbee and Alabama Rivers (watershed area: 51,921 and 58,896 km^2^, respectively). Their mean river discharge is nearly the same and together form the fourth largest coastal river discharge in the continental United States (1,866 m^3^s^−1^; Dykstra & Dzwonkowski, 2021). River discharge ranges from almost no flow (100 m^3^s^−1^), making it like a tidal lagoon, to large events (15,000 m^3^s^−1^) with magnitudes exceeding low flow on the neighboring Mississippi River (Dzwonkowski et al., 2018).

On the marine side, in the Gulf of Mexico, the natural period of oscillation is near resonance for diurnal tides, which nearly cancels semidiurnal harmonics and makes tides near the Main Pass of Mobile Bay strongly diurnal (form factor > 10; Figure 2). The largest harmonics near Main Pass, K1 and O1, have equivalent velocity amplitudes and nearly equivalent water level amplitudes (13.4 and 14.3 cm, respectively; Seim et al., 1987), which creates a fortnight tropic‐equatorial cycle (herein spring‐neap) ranging ∼0–60 cm. When the largest harmonics cancel, they form *dodge* tides as seen in Spencer Gulf, Australia (Nunes & Lennon, 1986), where the system becomes almost nontidal during neap periods. The diurnal tides are affected by river discharge in Main Pass where ebbing flow pulses large estuarine‐river plumes into the Gulf of Mexico (Greer et al., 2018). A smaller fraction of Mobile Bay estuarine water exchanges through the other inlet, Pass aux Herons, into Mississippi Sound (0.36; Kim & Park, 2012).

**Figure 3:**
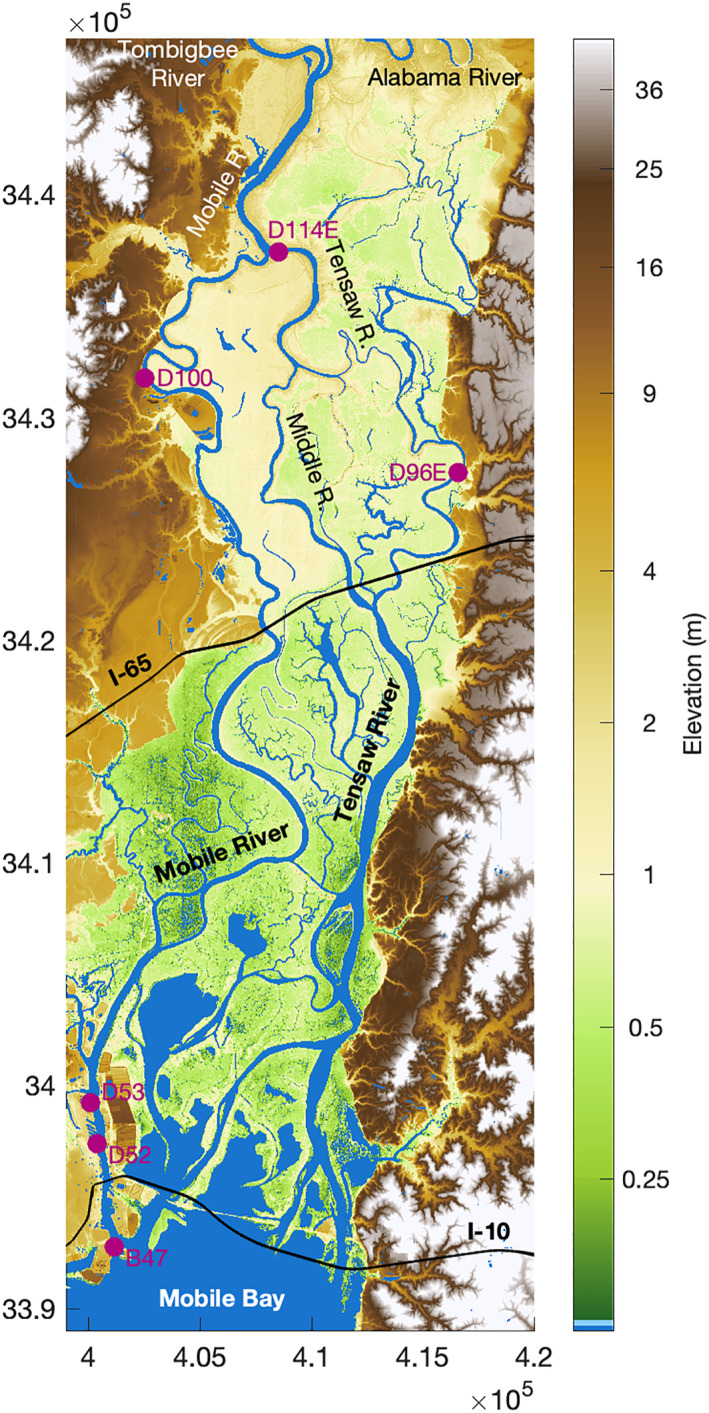
A map of the middle and lower Tombigbee‐Alabama Delta from the Tombigbee‐Alabama confluence to Mobile Bay. Axes units are in meters for UTM zone 16R.

At the bayhead is a delta (2,010 km^2^) with five distributaries called the Tombigbee‐Alabama Delta (Figure 3). The subaerial delta extends ∼95 km to the Suwanee‐Wiggins suture within a broad (10–20 km) pre‐Holocene valley (Greene et al., 2007). Four distributaries merge into the anastomosing Tensaw River, which has a local avulsion called Middle River that is notable for being wider and half the length of the parallel 24 km Tensaw reach. The other major distributary is the Mobile River, which extends along the west side of the delta from the port city of Mobile to the Mobile‐Tensaw Bifurcation (river kilometer (rkm) 112). The Mobile distributary is only 1 km shorter than the Tensaw mainstem, but generally receives two‐thirds of the river discharge and has higher water levels (Dykstra & Dzwonkowski, 2020a; Robinson et al., 1956). This lower delta region is known locally as the Mobile‐Tensaw Delta and is the most biodiverse location in temperate North America, earning a designation as the Mobile‐Tensaw River Bottomlands National Natural Landmark (Waselkov et al., 2016). The Mobile River continues 9.5 km to the Tombigbee‐Alabama River confluence (rkm 122). The landward single stem rivers have pronounced meander scrolls across the wide upper delta, suggesting strong fluvial influence and high morphodynamic activity (Smith, 1997). Because hydroelectric power peaking on the Alabama River (R238 E) created interference with tides in upstream reaches, the longitudinal transect of this study only continues up the Tombigbee River (Figure 2). The Tombigbee River extends beyond the delta apex (rkm 194) through a confined valley (∼1 km wide floodplain) to the downstream most point of flow regulation at the Coffeeville Lock & Dam (rkm 238).

### 3.1 A Quantification of System Geometry

**Figure 4:**
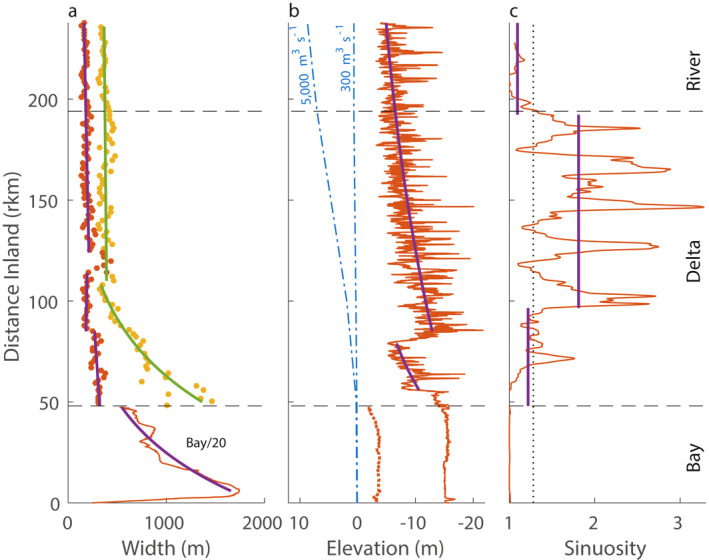
Geometry of the fluvial‐marine transition, including (a) width, (b) depth, and (c) sinuosity. The longitudinal transect of Mobile Bay‐Mobile River‐Tombigbee River (see Figure 2) is red and log fit with purple lines. Horizontal dashed lines (black) delineate the bay, delta, and non‐deltaic rivers. (a) In addition to the longitudinal width, the width of the Tensaw Distributary (including the Middle River) and Alabama River is added to capture a summed channel width (yellow dots, green line). To fit the wide bay, the bay width is divided by 20. (b) Depth is shown using thalweg elevation (NAVD88) and water levels (dot‐dash blue lines) for mean low‐flow (∼300 m^3^s^−1^) and near bankfull conditions (∼5,000 m^3^s^−1^). Thalweg elevations along the bay capture the dredged shipping channel, which is much deeper than the overall mean bay elevation (dotted red line). (c) Sinuosity is delineated as being negligible below ∼1.28 (dotted line; Lazarus & Constantine, 2013).

With single strand rivers, a large delta (2,010 km^2^; Dykstra & Dzwonkowski, 2020a), and a bay, the fluvial‐marine transition had large geometric changes in width, height, and sinuosity (Figure 4). The two inlet geometry has a combined width of 10 km and reaches 17m deep, which widens and shallows to the lower bay, geometry changes that only increase the landward area by 2 fold. For the entire bay, width convergence is much stronger than height convergence (L_w_ = 38 km; L_h_ ≈ 200 km) because the mean depth decreases only in the upper bay (Figure 4b). Due to the short longitudinal length of the inlets, their effect on the bay e‐folding lengths was limited.

At the bayhead, the delta channels are wide and deep (Figures 4a and 4b). The total channel width converges landward to the Tensaw‐Middle Bifurcation at rkm 107 (L_w_ = 41 km). Further landward, through the Mobile‐Tensaw Bifurcation to the dams, the total width is nearly constant (∼360m). Examining the Mobile and Tensaw channels independently shows nearly all width convergence is along the Tensaw channels (L_w_ = 33 km). The Mobile distributary width and depth significantly change further seaward (rkm 79 to 86), near the U.S. Interstate system I‐65 Bridge, quickly narrowing (mean of 300 to 190m) and deepening (mean of −6 to −13m). For low river discharge conditions, when water levels are low, height convergence is much stronger than width convergence to the bridge (L_h_ = 51 km; L_w_ = 210 km) and landward along the Tombigbee River to the dam (L_h_ = 159 km; L_w_ = 421 km). Using L_h_ and L_w_ to solve for area convergence (Equation 2) estimates the bay area e‐folding length as 31 km, the same as observing the bay area convergence directly (Figure S1 in Supporting Information S1), and is nearly the same as the estimate for the lower delta channels (L_a_ ≈ 30 km).

Like convergence, sinuosity also changed in the delta (Figure 4c). Along the Mobile and Tensaw Rivers, sinuosity exceeded 2.5 in some locations. The upper delta landward of rkm 96 was on average significantly higher (1.8; *p* < 0.01) than the other regions of the system. The elevated region of sinuosity reflects the commonly observed strait‐meandering‐strait planform of fluvial‐marine transition geomorphology (Dalrymple & Choi, 2007), and the sinuosity change occurring inland of a width and depth change is consistent with observations in tide‐dominated deltas (e.g., Fly, Yangtze, Irrawaddy; Gugliotta & Saito, 2019), even though the system is microtidal.

## 4 Data and Methods

### 4.1 Data Sources

Long‐term publicly available monitoring records were the original source of all data used (Figure 2, Table S1 in Supporting Information S1). Most water level, velocity, and river discharge data were accessed from Dykstra and Dzwonkowski (2020b) and updated through May 2020. The 21 stations used in this study were labeled with the first letter representing the body of water (e.g., G:Gulf, B:bay, D:delta, R:river) followed by the along channel distance inland from Main Pass along the longitudinal transect (i.e., rkm; converted from navigational river miles). Stations not on the longitudinal axis are to the east and are noted with an E (e.g., D96 E). Data from D100 and stations landward were accessed from the USGS (waterdata.usgs.gov/nwis) while the stations seaward were accessed from NOAA (tidesandcurrents.noaa.gov) and the Alabama Real‐Time Coastal Observing System (ARCOS; arcos.disl.org) except for B8, which was from the USGS. Most stations had a sampling interval between 6 and 60 min.

All water levels were referenced to the common vertical datum, NAVD88 (North American Vertical Datum 1988). Current velocities were determined from acoustic Doppler current profilers (ADCP) orientated for vertical (G‐14, B23) or across‐channel (B47, D53, D100) profiles, and from a benthic acoustic stress sensor (B8). To minimize differences between collection methods for the horizontal and vertical oriented ADCPs, only bins between one‐quarter and one‐third of the upper water column were used. This vertical bin range overlaps with the index velocity of the horizontal sensors as determined by the source organization (e.g., Ruhl & Simpson, 2005). The primary flow axis was determined by the major tidal harmonic axis (K1 & O1) with t_tide (Pawlowicz et al., 2002). For the Gulf of Mexico station (G‐14), the major tidal axis was in line with the shipping channel, following the longitudinal axis. Because the extensive data were averaged (described below), non‐tidal shelf circulation was not removed (e.g., inertial oscillations). While some data do not temporally overlap, all measurements could be referenced to discharge during a period without bathymetric changes in dredging or the construction of bridges or upstream dams. Specific details of data sources, length of records, and sensors used at each station is in the supplemental material (Table S1 in Supporting Information S1).

Local ground surface elevations were taken from USGS 3DEP Lidar (USGS, 2016, 2020), The Shuttle Radar Topography Mission (Farr et al., 2007), a NOAA DEM (NOAA National Geophysical Data Center, 2009), and Dykstra and Dzwonkowski (2020b). For topography, Lidar was converged to a 10m DEM using Opentopography. For bathymetry, the NOAA DEM was used to analyze the bay geometry but had a limited spatial extent and missing data in the mid delta. Thus, landward of the bayhead, depth, and area of the Mobile and Tombigbee Rivers were calculated using a DEM of the longitudinal channel from Dykstra and Dzwonkowski (2020b). No comparable data of the Tensaw could be acquired. Channel width was measured every 2 km and included all five major anastomosing channels of the Tensaw distributary (i.e., Tensaw, Blakely, Apalachee, Raft, and Middle Rivers). Because the Middle River regional avulsion was half the length of the Tensaw, Middle River widths were measured at a 1 km interval. Sinuosity was calculated by dividing the centerline length by the length of a low‐passed centerline using a 10km‐moving mean, the bin size revealing the highest mean sinuosity.

### 4.2 Timeseries Analysis

The water level and velocity timeseries for each station were analyzed for changes with river discharge and compared to reveal spatial variability, shown in river discharge‐longitudinal space. Because tide‐river interactions along a longitudinal transect are nonconcurrent, for each station, river discharge was temporally offset from upstream observations following Dykstra and Dzwonkowski (2020a), and tidal observations were related to the mouth (B0) after being offset by the observed lag time (see continuous wavelet transformations below). Capturing longitudinal changes from river discharge was first done to delineate the estuary and tidal river using methods based on water level or velocity observations (e.g., Hoitink & Jay, 2016). Neap and spring periods were determined from B0 tidal amplitudes (i.e., K1‐O1 beat) using the first and fourth quartiles, respectively. After being temporally lagged for each station, the lowest water level for every neap and spring period was identified. Instead of bulk averaging the entire timeseries, they were sorted by river discharge and averaged using a moving‐mean. Because station timeseries were different lengths, the bin size was determined using 10 degrees of freedom (i.e., bin = n/10, where n is the number of observations). Then spring means were subtracted from neap means. Results from each station were linearly interpreted longitudinally, revealing changes in discharge‐longitudinal space. The velocity method found the flood limit where the peak spring flood was zero. Peak spring floods were found by first identifying velocity amplitudes at each station using band‐passed time series (3–40‐hr Lanczos filter; e.g., Dzwonkowski et al., 2015), for amplitudes > 2 cm, periods of 24.8 ± 2 hr, and removing outliers (<p_25_ − 1.5p_r_ or > p_75_ + 1.5p_r_, where p_25_ and p_75_ are the 25th and 75th percentiles, respectively and p_r_ = p_75_ − p_25_; Figure S1a in Supporting Information S1). The corresponding non‐filtered values were the peak flood velocities (Figure S1b in Supporting Information S1). To estimate spring flood velocities, the sinusoidal spring‐neap relationship was utilized as sinusoidal functions have standard deviations $\sigma \left(\eta \right)=\eta /\sqrt{2}\approx 0.71\eta$ (η is amplitude), allowing $\bar{\eta }+\sigma$ to represent the 71st percentile. After being sorted by river discharge, moving‐means and moving‐standard‐deviations were taken of peak flood velocities using a window size of one month (31 days), and added (Figure S1b in Supporting Information S1). Like the water level method, results from each station were linearly interpreted longitudinally.

Continuous wavelet transformation was used to detect relative tidal amplitudes and relative tidal phases in a time‐frequency domain (Jay & Flinchem, 1997). Following previous tidal applications, the toolbox of Grinsted et al. (2004) was utilized with a Mortlet‐type wave and a non‐dimensional frequency scale of 6 (e.g., Sassi et al., 2012). This method calculates energy as a wavelet power spectrum, wavelet cross correlation between stations, and significance for both at a 95% confidence interval. Because the long timeseries of water level measurements at the mouth (D0; 38 years) were concurrent with all other observations, water level and velocity were normalized to the mouth water level observations. This was done using the wavelet cross correlation to capture relative phase in the water level and velocity. For relative amplitude, the relative phase was used to offset the significant wavelet power spectrum of each station before dividing it by the wavelet power spectrum from the mouth.

For the tidal phase lag (ϕ; i.e., phase between velocity and water level), a wavelet cross correlation was conducted where nearby water level and velocity measurements were available. Because the phase lag near the mouth (B8) was stationary (relatively insensitive to river discharge), the velocity phases relative to D0 water level were corrected with a constant offset. The offset was based on the phase lag (79.8°, 5.50 hr) of near surface currents (−4m) in Main Pass from a short deployment (∼3 months). For the local wavenumber (k), the wave frequency was divided by the celerity (c), calculated from the phase difference between two stations (i.e., k = ω/c).

To identify the effects of river discharge on tides, amplitude, phase, phase lag, and wavenumber timeseries were sorted by river discharge and averaged using a moving‐mean of at least 10 degrees of freedom. At very high and low river discharges data was insufficient to complete averages, minimizing potential error. This approach was used instead of applying a bin of the same size because datasets of different lengths can cause longer datasets to show more variability with river discharge than the shorter datasets. Additionally, binning by river discharge was not conducted because the error would vary for every bin.

## 5 Results

### 5.1 Delineating the Estuary and Tidal River Using Tidal Variability

**Figure 5:**
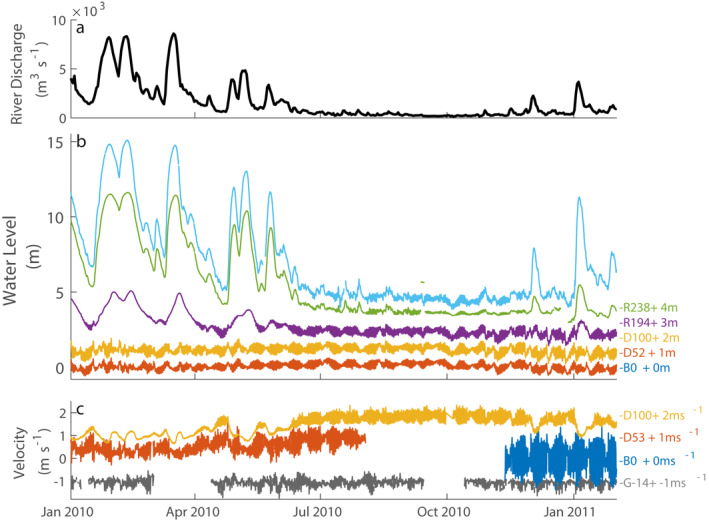
Time series of (a) discharge, (b) water level, and (c) near surface velocity in 2010 showing tidal variability throughout the Alabama coastal region (Figure 2). For b and c, the measurements at each station are spaced by one unit and labeled on the right. Neighboring stations or coupled instrumentation have the same color. (c) For velocity, positive values indicate landward flow.

Within the convergent geometry, water level and velocity throughout the system were primarily influenced by tides and river discharge, generating notable variability in the time series (Figure 5). To demonstrate the variability, we focus on the year 2010, a representative year with several discharge events in the winter and spring followed by a dry summer and fall (Figure 5a). River discharge ranged 130‐8,600 m^3^s^−1^ with large events lasting 1–4 weeks. Near concurrent water level peaks for the landward most observations captured the effects of terrestrial runoff events (R194, R238), which became smaller further seaward (e.g., D100; Figure 5b). At the seaward most stations (B0 and D52), water level variability was mostly <1m with fortnight modulation, indicating variability was primarily tidal. When river discharge and water levels were low, a fortnight tidal modulation was visible at all stations, even at R194 where tidal amplitude was smallest.

Velocity also shows the influence of both discharge and tides. Discharge events caused strong non‐tidal seaward flows at the most landward velocity station (D100). Further seaward, however (B0, D53), velocity variability was primarily tidal with fortnight modulation, reflecting water level dynamics and shelf currents (G‐14). When river discharge was low, bidirectional tidal currents were observed at all stations. When river discharge was high, the fortnight modulation appeared noisier, particularly for velocity, compared to water levels (e.g., D53 vs. D52).

**Figure 6:**
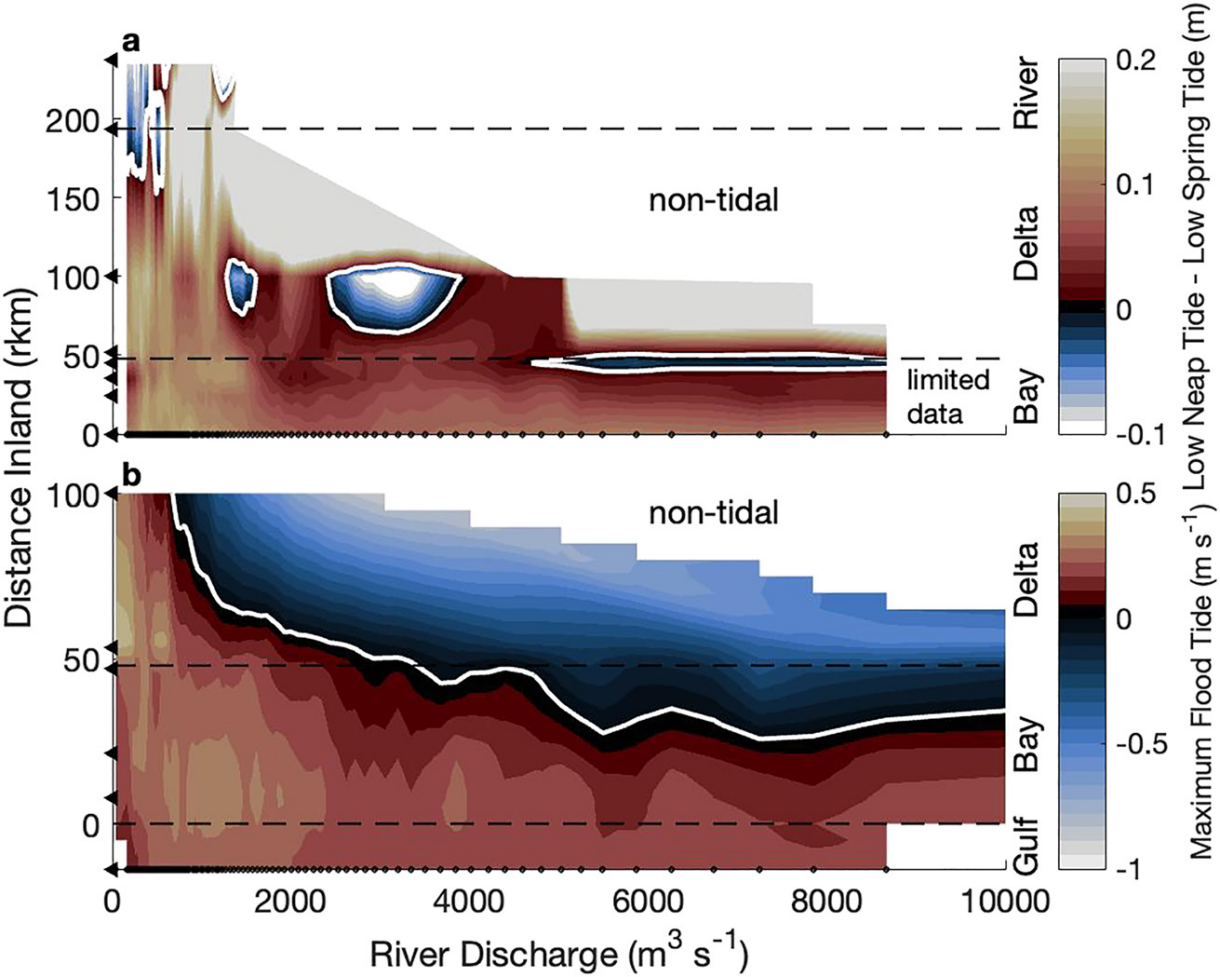
The estuary‐tidal river boundary is delineated by discharge conditions using (a) the difference between lowest neap water levels and lowest spring water levels (e.g., Hoitink & Jay, 2016) and (b) the maximum tidal velocity produced by the flood tide. Positive (seaward flow) values suggest estuarine conditions (red/yellow), negative values suggest tidal river conditions (blue), and the delineated boundary is a white line. The *y*‐axis of each subplot shows station locations (triangles) and has different scales, while the *x*‐axis shows bin spacing of river discharge levels (dots, *n* = 101). The white space is either non‐tidal or had insufficient data for the analysis.

To identify the location and extent of tide‐river interactions we adopt the approach of Hoitink and Jay (2016) for river discharge conditions ranging three orders of magnitude. At low discharge (Q_r_ < 300 m^3^s^−1^), low neap water was much higher than low spring water (>10 cm) in most of the system, suggesting estuarine conditions, except far inland where low spring water was higher (Figure 6a). The switch occurred near the delta apex and is interpreted as the estuary‐tidal river delineation. As river discharge increased, low spring water became similar to low neap water and then higher, switching in the middle of the delta at 1,300 m^3^s^−1^ and bayhead at ∼5,000 m^3^s^−1^. However, some areas switched multiple times. A careful examination of the data did not reveal any artifacts, suggesting the method was inconsistent and unreliable.

Instead of using water level to delineate the estuary and tidal river, a different approach used velocity to delineate bidirectional and unidirectional flow by finding the flood limit with maximum velocity (Figure 6b). At low discharge, maximum velocities were positive at all velocity stations and were strongest in the lower delta. As discharge increased, flood velocities decreased in the middle delta until the maximum velocity reached 0 ms^−1^ at 600 m^3^s^−1^, detecting the flood limit (Figures 6b and S1b in Supporting Information S1). At this discharge, the maximum flood velocity began decreasing at the bayhead and increasing in the lower bay. At a higher discharge (∼2,000–3,000 m^3^s^−1^), the flood limit was detected at the bayhead and the flood velocity became smaller across the bay, allowing the flood limit to reach the middle of the bay at high discharge (>5,000 m^3^s^−1^). These sequential observations and interpolations capture a consistent seaward shift of the flood limit with discharge, unlike the water level method (Figure 6a). Interestingly, at the median discharge (∼1,000 m^3^s^−1^), the flood limit was at the major geometry transition of the Mobile Distributary (∼rkm 80), where the system transitioned from being wide and shallow to narrow and deep (Figures 4 and 6b).

### 5.2 Tidal Response to Discharge

#### 5.2.1 Tidal Amplitude and Lag Time

**Figure 7:**
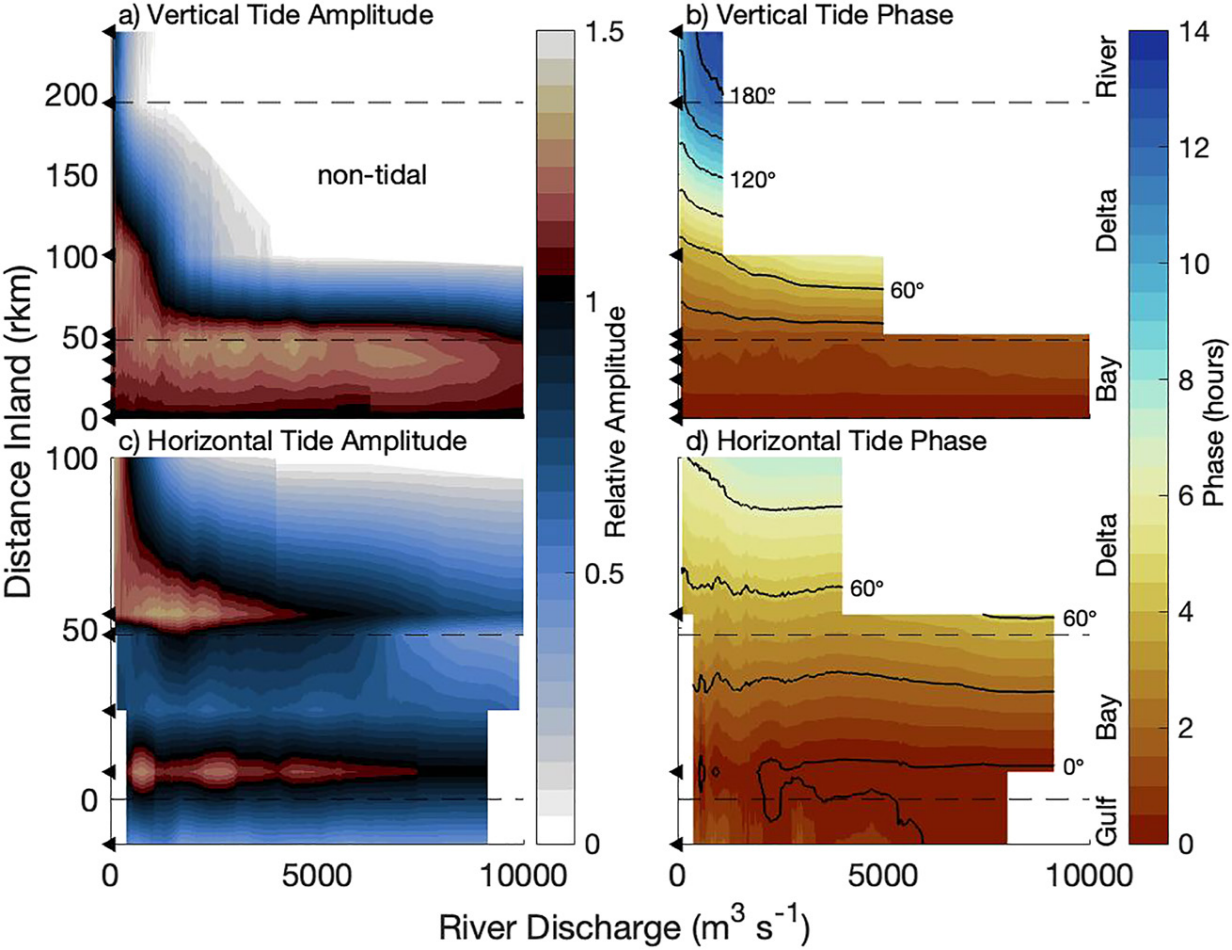
Diurnal tide observations in longitude‐river discharge space of the tidal (a, c) amplitude and (b, d) phase of (a, b) the vertical tide observed using water levels and (c, d) the horizontal tide observed using near surface velocity. Amplitudes and phases are normalized to the estuary mouth to show changes longitudinally. The layout of each subplot follows Figure 6.

The seaward shift of the flood limit by discharge suggests a dynamic tidal response, which is further investigated, first using water level observations to capture the vertical tide. Assuming the estuary is ideal, strong landward convergence to the mid delta suggests the tidal amplitude would remain relatively constant to this location and decay landward. Instead, at low discharge, the D1 vertical tide (D1η) amplified to the mid delta, peaking at D100 at 1.5 times the amplitude of Main Pass (D1η_0_; Figure 7a). D1η attenuated across the upper delta and then amplified again from the delta apex to the dam, reaching similar amplitude to D1η_0_, but 238 km inland. As discharge increased, the most inland reaches at the delta apex and dam (R194, R238) quickly damped and became undetectable almost simultaneously. Further seaward, the general trends show river discharge caused D1η to first amplify and then attenuate. Peak amplitudes for the middle of the delta occurred at ∼600 m^3^s^−1^, for the bayhead at ∼2,000–5,000 m^3^s^−1^, and at higher discharges, for the middle of the bay. Interestingly, peak amplitude for each region occurred further seaward as discharge increased.

Given the large amplitude changes with discharge, tidal phase and celerity are also investigated, using a lag time relative to Main Pass (i.e., travel time from B0; Figure 7b). General trends show D1 vertical tide lag times (D1η_t_) increased in a landward direction, though the distance traveled each hour was not consistent. At low discharge, the lag time to the bayhead, delta apex, and dam (rkm 48, 194, and 238, respectively) was approximately 1, 11, and 11.5 hr, respectively, showing wave celerity (i.e., dxdt^−1^) was very fast across the bay and slowed across the delta before accelerating further inland. This also indicates the entire system captures approximately half the D1 wavelength. As discharge increased, general trends show lag times increasing. The strongest responses to river discharge were at the most landward reaches where lag times increased, shifting from 11–11.5 to 13–14 hr, at which point the tidal signal was attenuated. The bayhead and bay had weaker responses with notable lag time increases of ∼30 min at 5,000 and 8,000 m^3^s^−1^, respectively.

The vertical tide response to discharge was similar to the response of the horizontal tide. The diurnal amplitudes of the horizontal tide (D1_u_) were largest near Main Pass and the bayhead, where the flow was constricted (Figure 7c). As discharge increased, D1_u_ quickly decreased at the furthest inland velocity site, in the middle delta, faster than the vertical tide and without first peaking. D1_u_ increased at the bayhead, peaking at ∼2,000 m^3^s^−1^, and then decreased at higher discharge. Observable damping also occurred near the mouth and at G‐14, 14 km offshore in the Gulf of Mexico. Compared to D1η, D1_u_ was more spatially variable and damped at a lower discharge.

The vertical and horizontal tidal components also showed some different lag time responses to discharge (Figure 7d). The diurnal horizontal tide lag time (D1_ut_) increased seaward and with discharge like D1η_t_, but had longer lag times. This is most clearly seen at the bayhead, where the lag time for D1_ut_ was approximately three times that for D1η_t_, indicating the celerity was a third of D1η_t_. At low discharge in the Gulf of Mexico (G‐14), the currents lagged the mouth by ∼1.5 hr, suggesting the surface currents were strongly influenced by estuarine exchange (e.g., tidal rectification of an inlet altering flow directions). As river discharge increased, the lag time decreased at G‐14 and was nearly in phase with the mouth when discharge exceeded 2,000 m^3^s^−1^, indicating the effects of river discharge on tidal dynamics extended onto the shelf.

#### 5.2.2 Tidal Wave Propagation‐ Wavenumber and Phase Lag

**Figure 8:**
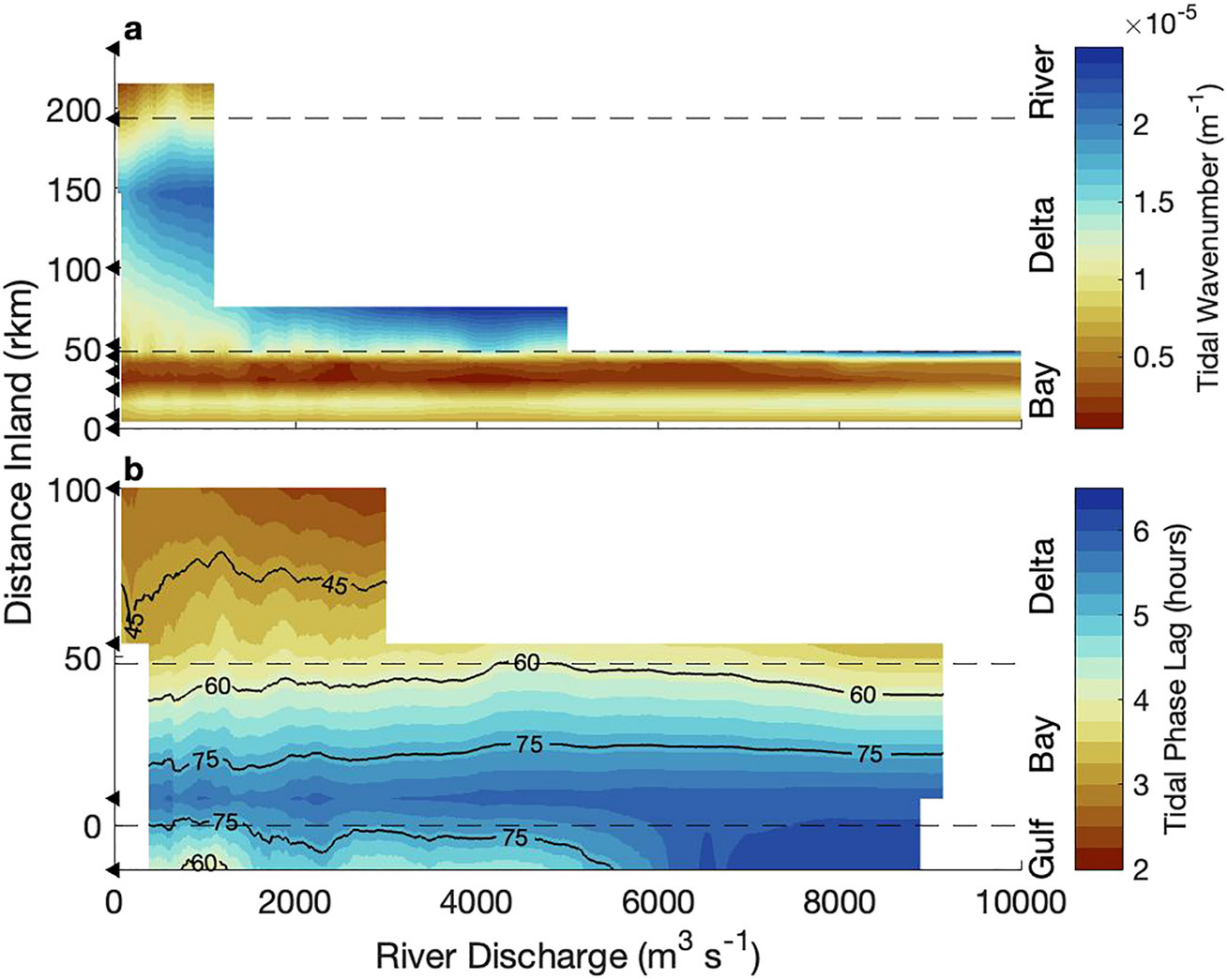
The propagation of diurnal tidal waves is captured using (a) wavenumber and (b) phase lag ($D1_{\phi }$) in longitude‐river discharge space. (b) Black contours show degrees. The layout of each subplot follows Figure 6, except for the wavenumber values in (a), which were resolved at the midpoint between stations.

The spatial variability of tidal response to discharge suggests there were changes in the waveform, commonly determined by frequency, wavenumber (i.e., change in wavelength), and phase lag ϕ. Because forced tidal waves have consistent frequency determined by astronomical processes, we focus on changes in wavenumber and phase lag ϕ. First, examining the spatial variability of the D1 wavenumber, using the vertical tide, general trends show the wavenumber across the bay increased and then decreased, similar to the width (Figure 8a). Across the delta, the wavenumber also increased and then quickly decreased as the tide approached the dam, nearly matching the large upper bay values. As discharge increased, general trends show the wavenumber increased. One exception was in the bay, where the wavenumber had a small decrease with discharge to 2,000 m^3^s^−1^, but above 5,000 m^3^s^−1^, the wavenumber increased with discharge. This general trend suggests the wavelength decreased landward and with river discharge, ranging from over 2,000 km in the bay at low discharge to as small as 300 km as high discharge damped the tide in the delta.

Responses to discharge in the D1 phase lag ($D1_{\phi }$) were evident in the differences of D1_ut_ and D1η_t_ and are clearly captured in Figure 8b. Near Main Pass, at low discharge, near surface tidal currents phased 5.7 hr ahead of the water levels, or 82°. This $D1_{\phi }$ decreased landward to 2.7 hr (39°) in the middle of the delta. As discharge increased across the delta and bay, $D1_{\phi }$ increased, peaked, and decreased, ranging almost an hour (12–15°). At the Gulf of Mexico site (G‐14), $D1_{\phi }$ greatly increased when discharge exceeded 5,000 m^3^s^−1^, reaching 6.2 hr (90°), and did not peak. Interestingly, peak $D1_{\phi }$ was detected in the middle and lower delta at approximately the same river discharge the flood limit was detected and the tidal amplitudes peaked.

## 6 Discussion

The small diurnal *dodge* tides traveled 238 km from the Gulf of Mexico to the Coffeeville Lock & Dam, forming the Tombigbee‐Mobile Bay fluvial‐marine transition. Even though the tidal amplitude could be the same at both ends of the transition, like the Hudson River (Ralston et al., 2018), along the transition, the waveform (amplitudes, lag times, phases, wavenumbers) was strongly modulated by geometry and river flow. The wide range of river discharge, spanning several orders‐of‐magnitude, allows us to generally separate tidal modulation by geometry from river effects in the following section, before focusing on reaching specific feedbacks. We then identify broader applications of this study to other systems, other methods, and the general interactions of tides, rivers, and channel geomorphology.

### 6.1 Longitudinal Shift of the Tide‐River Interactions

#### 6.1.1 Geometric Controls on Tidal Waves

For geometric effects on tides, the cross‐sectional area gradually decreased in a landward direction with sudden changes in the width‐depth ratio, forming distinct convergent environments. In the bay, convergence was primarily controlled by the width (L_a_ = 31rkm). Despite the shallow environment, the fast‐amplifying tidal wave and the associated phase lag suggest the bay is hypersynchronous. In the lower delta, width and height convergence together formed approximately the same area convergence as the bay (L_a_ ≈ 30rkm). The relatively consistent amplitude and wavenumber suggest the reach is *ideal* with partial reflection and friction nearly balanced. Further landward was a backwater environment with weaker convergence primarily controlled by the height (L_a_ = 115rkm). The slow attenuating tidal waves suggest the reach was hyposynchronous, except near the end where tidal waves may have reflected off the dam.

#### 6.1.2 River Discharge Controls on Tidal Waves

**Figure 9:**
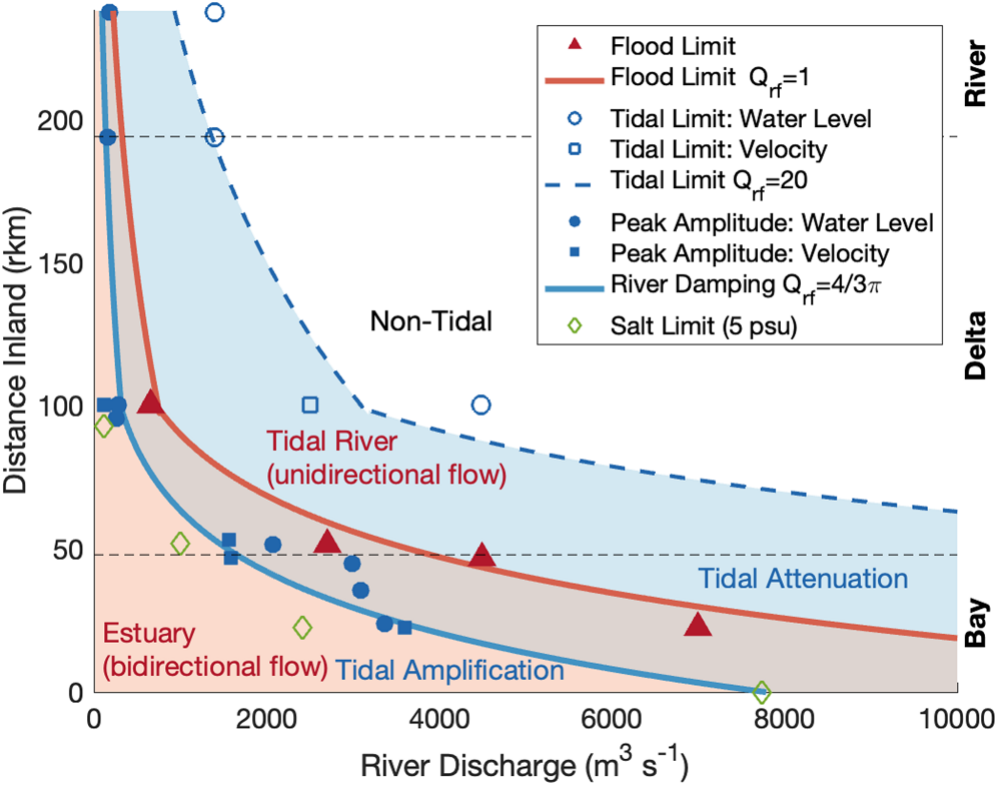
Observations (symbols) and solutions (lines/fill) summarizing tide‐river dynamics along the fluvial‐marine transition. The primary effects of river discharge on tides are colored for flow direction (red) or amplitude (blue) and gradually shift seaward as river discharge increases. The red fill captures the longitudinal region with the bidirectional flow and is the tidal estuary, while the region without red fill is unidirectional and a tidal river where tides persist. The blue fill captures the longitudinal region where river flow attenuates tides and is contrasted with the seaward region where river‐induced stratification amplifies tides and the landward region where tides are damped. The overlapping red‐blue region captures the low energy area where reversing tides attenuate river flow. Symbols represent conditions observed at individual stations. The flood limit was approximated using a Q_rf_ value based on Figure S2a. The 5 psu salinity limit is from Dykstra and Dzwonkowski (2020a, 2020b) when river discharge is increasing and remains just seaward of river damping and peak tidal amplitudes.

Similar to the geometric modulation of the tidal wave, the waveform was also modulated by river discharge. The most consistent river modulation led to tidal phases (i.e., lag time from D1) and wavenumbers increasing with river discharge, indicating wave celerity and wavelength decrease with river flow, supporting analytical theory (e.g., Jay & Flinchem, 1997; Kästner et al., 2019). The tidal amplitude and phase lag had two responses to river discharge, increasing and decreasing, that depended on the river discharge magnitude. To clearly identify how river discharge affects tidal amplitude, the river discharges at which the tidal amplitude peaks and damps are identified for each station (Figure 9). As discharge increased, each station had a common response: the tide first peaked in amplitude, followed by the flood limit, and then at higher discharges, the tide damped at the inland stations. Comparing stations longitudinally shows the pattern shifts seaward or landward as a function of discharge with high (low) discharge shifting responses seaward (landward).

The location of the flood limit and peak tidal amplitude closely followed our analytical solutions based on convergence length (Equation 6b). Because area convergence changed in the middle of the delta, different solutions were used for seaward (L_a_ = 31 km, rkm 6–86) and landward reaches (L_a_ = 115 km, rkm 86–238; Figure 9). All four flood limit observations were within 10 km of the analytically solved location for *Q*_rf_ = 1, delineating the bidirectional estuarine currents (red shading) from the unidirectional tidal river. The peak tidal amplitude closely aligned with the analytically solved location for *Q*_rf_ = 4/3π, delineating the landward region where river discharge would be expected to begin attenuating the tide (blue shading; Kästner et al., 2019). This delineation also aligns well with the conditions at which river discharge is observed by Noble et al. (1996) to begin increasing subtidal bottom currents and bed friction (3,000 m^3^s^−1^, rkm 25). Deviations from the theoretically predicted locations of flood limit and peak tidal amplitude in the upper bay are likely a result of the bay being hypersynchronous and not having *ideal* geometry. Despite the non‐*ideal* geometry, which likely caused peak amplitude to remain near the bayhead for a wide range of river discharges (Figures 7a and 7b), the solution captured the effects of river discharge well at individual stations (Figure 9, filled blue symbols).

By additionally including the observed salinity limit (5 psu), we see it also moves seaward with river discharge and follows *Q*_rf_ = 4/3π, suggesting the solution also captures estuarine flushing. By assuming some level of stratification seaward of the salinity limit, the *Q*_rf_ = 4/3π point in the system appears to be the location at which the primary river discharge effect on tides is reducing friction through stratification, allowing the tides to amplify. Landward of this point, river discharge would be expected to attenuate tides due to increased friction from strong flow velocities.

The simple analytical equation based on tide‐geomorphic equilibrium (i.e., e‐folding length) and conservation of mass captures tide‐river interactions shifting seaward as river discharge increases (i.e., Equation 6b; Figure 9). For the weaker convergence of the inland backwater reaches, tidal‐fluvial processes (e.g., tidal rivers) moved seaward much faster with river discharge. Within a constant region of area convergence, river discharge does not change the longitudinal length of any given tide‐river interactions as they shift seaward (Figure 9). For example, the region where tides reverse and attenuate from river discharge does not change in size longitudinally (red‐blue shading). This low energy region, which typically contains the turbidity maximum, can be difficult to identify because the tidal dynamics are poorly captured in analytical solutions (Figure 1c; Burchard et al., 2018; Jay & Flinchem, 1997; van Rijn, 2011). In Mobile Bay at rkm 20, a turbidity spike is observed when Q_r_ > 5,000 m^3^s^−1^ (∼0.01–1 g/L; Ha & Park, 2012) and matches the predicted region of the turbidity maximum, suggesting the simple solution may also be able to estimate the longitudinal shifts of estuarine sediment dynamics.

### 6.2 Reach Specific Tide‐River‐Geomorphic Interactions

#### 6.2.1 The Transition From Tide to River Dominated Morphology

In the Mobile‐Tombigbee fluvial‐marine transition, the change from tidal‐dominated to river‐dominated morphology was clearly identified in one region. Assisted by a plan view (e.g., Figure 2), the transition from river to marine‐dominated environments is commonly delineated at the bayhead with tidal influence reaching the Mobile‐Tensaw bifurcation (e.g., Byrnes et al., 2010). Using width, depth, and area, our results suggest the bayhead transition to the delta does not significantly affect the total cross‐sectional area or area convergence when the non‐channel regions are included (Figure 4, S1 in Supporting Information S1). Instead, the fluvial‐marine geomorphic change is ∼40–50 km inland of the bayhead and seaward of the Mobile‐Tensaw bifurcation (∼rkm 90–100).

The tide‐dominated and river‐dominated geometries spatially coincided with their reflective processes when the flood limit was at the geomorphic change. Under these conditions, in the lower delta, the tidal dynamics suggest the reach is *ideal* and in morphodynamic equilibrium, aligning well with geomorphic theory (e.g., Dalrymple & Choi, 2007; Gugliotta & Saito, 2019). However, the hydrodynamic observations show this only occurred at median river discharge conditions (i.e., ∼1,000 m^3^s^−1^) as the flood limit shifted longitudinally with river discharge (∼±80rkm). Even though a system like the Mobile‐Tombigbee fluvial‐marine transition may have characteristic geomorphology, the hydrodynamics may be highly variable and difficult to approximate for any given time using only the geometry.

#### 6.2.2 The Height Convergent Backwater

Landward of the sharp geomorphic change is a long backwater reach with weak height convergence where strong friction damped the tides before amplifying from dam reflection. At low discharge, results suggest the height convergence extended tidal influence inland. Because of the smaller upstream cross‐sectional area, a small increase in river discharge quickly attenuated the tide. Kästner et al. (2019) shows this is done directly by increasing friction and indirectly by increasing the water surface slope, reducing convergence. Reduced convergence in the backwater reaches of the Tombigbee River and the Mobile distributary, which was also height convergent, occurred with river discharge increasing the water surface slope while the bed remained stationary (Figure 4b).

While height may converge more than the width in backwater reaches, a different lateral component can be critical, sinuosity. Sinuosity attenuates tides through flow separation around bends, inducing form friction (Bo & Ralston, 2020), which is not accounted for in analytical tide models (e.g., Buschman et al., 2009; Kästner et al., 2019; van Rijn, 2011). The processes controlling sinuosity remain highly debated (e.g., ; Hoitink et al., 2017; Lazarus & Constantine, 2013; Schumm & Khan, 1972). Using planform observations, sinuosity is associated with peaking near the estuarine turbidity maximum, seaward of the flood limit, because of the large sediment availability (e.g., Choi et al., 2020; Leuven et al., 2018). Using planform and depth observations, we show sinuosity increased just landward of the geomorphic change (rkm 96) and coincides with the backwater reach, similar to other systems (e.g., Fly, Yangtze, Ganges‐Brahmaputra, Irrawaddy; Gugliotta & Saito, 2019). Because backwater reaches have relatively low availability of medium to coarse grained sediment (Ensign & Noe, 2018; Nittrouer & Viparelli, 2014), sinuosity in the fluvial‐marine transition is likely controlled by flow deceleration, as presented theoretically by Lazarus and Constantine (2013). Thus, backwater dynamics that increase height convergence and reduce tidal attenuation also increase sinuosity and form friction, which are two factors that may balance in backwater reaches.

Another complication for backwater analytical theory is dam reflection. Analytical theory using a constant convergence predicts dam reflection amplifies tides and decreases their phase (van Rijn, 2011), as observed in the Ems and Guadalquivir Estuaries (Díez‐Minguito, Baquerizo, Ortega‐Sanchez, Ruiz, & Losada, 2012; Talke & de Swart, 2006) and shown here on the Tombigbee River when river discharge was low. Theory indicates the reflected wave becomes negligible beyond half a convergence length (Friedrichs, 2010); suggesting dam effects are minimal seaward of rkm 180. For R194, slow wave celerity approaching this station suggests the incident and reflected waves were more than a quarter out of phase and reduced the tidal amplitude (Figure 7b). As river discharge increased, the tide attenuated and phase increased, like further seaward (Figure 7), suggesting the tide‐river interactions did not greatly change the dam effects. Because the local bed slope is relatively flat (Figure 4b), suggesting the backwater effects were small, a future investigation is warranted for dam effects in reaches with strong backwater effects.

### 6.3 Broader Applications

The tide‐river‐geomorphic channel interactions of the Tombigbee‐Mobile fluvial‐marine transition provide many insights that are broadly applicable. The diurnal microtidal system followed the theory developed for semidiurnal and mixed tidal environments with larger tidal ranges. For example, the theories of friction and width convergence are based on the dominant tidal specie and can explain D_2_ and the D_4_ overtide amplitudes and phases in semidiurnal environments, like the Columbia, Frasier, and St. Lawrence, but not D_1_ (Godin, 1985; Jay & Flinchem, 1997). Demonstrating the D_1_ tidal amplitude, phase, and wavenumber followed expected theory in a diurnal system provides substantial support for friction being controlled by the dominant tidal specie. This aligns with the theory of M2 transferring energy to K1 and O1 to explain the insensitivity of D_1_ to river discharge in semidiurnal environments (Godin, 1985, 1999). The opposite may also be true in diurnal environments, but this has yet to be observed. The observations presented here may be the first diurnal tide observations to capture tide‐river and tide‐channel geomorphic interactions, highlighting an unequal portion of studies in semidiurnal systems.

In well‐studied semidiurnal systems, observed longitudinal shifts have only been identified to a limited extent, but generally show that distance increases with the relative range of river discharge (Table 1). The large flood limit shift and range of river discharge in this study exceed St. Lawrence, Yangtze, and Columbia Rivers. The variability supports Equation (6a), (6b), (6c), indicating that for every order of magnitude increase in river discharge, the flood limit and other tide‐river interactions shift seaward twice the e‐folding length. Deviations may result from river regulation, such as the Guadalquivir River where ceasing all river discharge allows the flood limit to approach the regulating dam (Díez‐Minguito, Baquerizo, Ortega‐Sanchez, Navarro, & Losada, 2012; Díez‐ Minguito, correspondence), or from channelization, such as the Hudson River where dredging reduced inland convergence while increasing the tide‐river energy ratio, competing factors for the flood limit location that may have increased or decreased the overall shift, respectively (Ralston & Geyer, 2017; Ralston, correspondence).

**Table 1:** Longitudinal Shifts of the Flood Limit Observed in Various Systems and Their Associated Range of River Discharge

Fluvial‐marine transition	Flood limit longitudinal shift km (locations)	River Discharge Range order of magnitude (m^3/s)
St. Lawrence River^a^	∼38 (Grondines to Deschambault)	0.7 (7,000–32,000)
Yangtze River^b^	∼85 (Zhenjiang to Jiangyin)	0.9 (∼10,000–80,000)
Hudson River^c^	∼90 (Troy to near Tivoli)	1.6 (∼100‐4,400)
Columbia River^d^	∼110 (St. Helens to Astoria)	1.1 (1,800‐24,000)
Guadalquivir River^e^	110 (Alcalá del Río dam to Port of Bonanza)	‐‐ (0–3,000)
Tombigbee River‐Mobile Bay^f^	∼180 (Jackson to Middle Bay Lighthouse)	2.2 (80‐15,000)

^a^ Matte et al. (2014).

^b^ Guo et al. (2015); Shen (2003).

^c^ Ralston and Geyer (2017); correspondence.

^d^ Jay et al. (2015); Jay, correspondence.

^e^ Díez‐Minguito et al. (2012a) correspondence.

^f^ This Study.

In a natural alluvial system, convergence and tidal energy reach a morphodynamic equilibrium which regulates the flood limit location. Because tidal energy scales with the damping rate, in a landward direction, tides with more energy attenuate faster (e.g., larger amplitudes, shorter periods; Equation (3a), (3b)) and should theoretically cause convergence to scale with tidal amplitude. However, empirical investigations have found limited support (e.g., Davies & Woodroffe, 2010; Leuven et al., 2018; Savenije, 2012) and may be a result of grouping all systems regardless of tidal period, which Equation 5 suggests is critical to morphodynamic equilibrium. For example, Savenije (2012) shows area e‐folding lengths can range greatly, ∼10–100 km, but separating the diurnal and semidiurnal systems, shows that mean area e‐folding lengths of the diurnal systems is more than twice the semidiurnal systems. Equation 5 also suggests diurnal systems have longer fluvial‐marine transitions (i.e., T^2^ ∝ L_p_), reflecting tide‐river theory and observations of diurnal tides attenuating landward of semidiurnal tides (e.g., Gallo & Vinzon, 2005; Godin, 1999). The longer and less convergent geometries of diurnal systems may also cause larger longitudinal shifts from variable river discharge in diurnal systems than semidiurnal systems (i.e., Equation (6a), (6b), (6c)).

Like tidal controls on morphodynamics, river discharge shapes the backwater geometry and may also indirectly affect the tide‐river longitudinal shifts. High river discharge has a relatively steep water level slope that extends further seaward with resulting currents that scour the bed, creating a deep region at the seaward most location of river‐dominated geometry (e.g., Figures 1b and 4b). Morphodynamic models indicate the range of river discharge controls the bed slope and backwater length (L_b_ = h/s, where s is bed slope; Ganti et al., 2019), subsequently increasing height convergence for tides. Thus, not only does a large river event force tide‐river interactions far seaward, it also excavates a deep height convergent channel for tides to intrude further landward during low river discharge. These patterns are consistent with proposed river scouring dynamics in the Tombigbee River‐Mobile Bay system (Dykstra & Dzwonkowski, 2020a) and suggest morphodynamic feedbacks in backwater reaches increase the range of tide‐river longitudinal shifts from variable river discharge.

Dynamic longitudinal shifts do not fit within traditional static definitions of tide‐river environments (e.g., *estuary*, Cameron & Pritchard, 1963) and require methods that capture their dynamics. The water level method of Hoitink and Jay (2016), capturing the subtidal setup, reliably delineate longitudinal shifts of the estuary‐tide river boundary on the Columbia and Hudson Rivers (Jay et al., 2015; Ralston et al., 2018). This method may be preferred to capture the flood limit with velocity measurements because water level measurements are more common, easier to quality control, and analyze. However, a primary process controlling subtidal setup, Stokes transport, is small in systems with a small tidal amplitude‐depth ratio or when highly stratified and can be complicated by tide‐river interactions (Jay, 2010; Sassi et al., 2012). All these factors describe our study site and likely explain why the first test of the methodology in a diurnal and microtidal system, shown here (Figure 6a), displayed inconsistent behavior. The method may not be suitable for low energy tidal systems and requires further analysis. The flood limit determined with tidal velocity observations was a more robust estuary‐tidal river delineation in this diurnal‐microtidal system and may be true for other systems. Not only does it reflect a primary delineation for analytical solutions (e.g., Jay & Flinchem, 1997; Kästner et al., 2019; van Rijn, 2011), it only requires a duration of one tidal cycle per river discharge level and is a general concept that can be more easily communicated to the broader scientific community.

Velocity is also critical for friction and discharge and is commonly converted from water level observations, but this process could produce sizable errors. The conversion assumptions ([a]: vertical tide represents horizontal tide, [b]: tidal wavenumber and phase lag do not change through time, and [c]: longitudinal consistency of river discharge, subtidal velocity, and tidal phase lag; Buschman et al., 2009; Sassi & Hoitink, 2013) are appropriate for pristine channels (i.e., constant width and height; Frederichs, 2010; Kästner et al., 2019) but may not be appropriate for real environments with convergence and river discharge, like the Tombigbee River‐Mobile Bay fluvial‐marine transition shown here. For the assumption of longitudinal consistency, some studies acknowledge spatial variability in river discharge but suggest errors in timing and magnitude should be minimal because river waves are much longer than tidal waves in the fluvial‐marine transition, pointing to the long period of river waves (e.g., Jay & Flinchem, 1997; Sassi & Hoitink, 2013). However, the river waves in the delta are slow, and when the system is still impacted by tides, river waves range from 350–1,060 km (Dykstra & Dzwonkowski, 2020a). This length is approximately the same as the tidal waves and not much longer than the fluvial‐marine transition, indicating river discharge was not spatially consistent. To account for the river wave propagation, river discharge was lagged for each station based on observations of the river waves (Dykstra & Dzwonkowski, 2020a), which produced results more consistent with theory. Due to the complexities of tide‐river interactions, particularly in backwater environments, simulating horizontal tidal waves from water level observations to calculate velocity, friction, and discharge may produce sizable errors.

Not only does river discharge affect tides, but the modulated tides can also affect river discharge itself, generating feedback. Such interactions traditionally show river discharge has negative feedback by either increasing friction through higher flow, which damps tides and generates subtidal setup, or reducing friction through stratification, which amplifies tides (Hoitink & Jay, 2016; Jay & Flinchem, 1997). This study suggests both processes can occur simultaneously and are interconnected with stratification amplifying tides in an estuary and passing more tidal energy inland, which can cause a larger subtidal setup in the tidal river. Thus, increasing the magnitude of river discharge could generate a larger subtidal setup and reduce the momentum of river discharge, further increasing the negative feedback of river discharge. Positive feedbacks also occur in the backwater reach through river friction increasing river setup and lowering convergence, causing tides to quickly attenuate.

## 7 Conclusions

Tide‐river‐geomorphic interactions are studied in the diurnal microtidal Tombigbee River‐Mobile Bay fluvial‐marine transition with the water level, velocity, and discharge observations, where the large range in river discharge make the system highly dynamic. From this study, the primary findings include:

1. Longitudinal shifts in tide‐river interactions (e.g., flood limit, tidal limit) are primarily controlled by the ratio of river discharge to tidal discharge and convergence, which, as river discharge fluctuated, shifted the flood and tidal limits ∼180 km
2. River discharge simultaneously amplified tides in seaward reaches while damping tides in landward reaches, passing more tidal energy inland where it attenuated faster
3. Diurnal tides amplified from convergence and dam reflection and attenuated where convergence was small, supporting the theory that tidally generated friction is created by the dominant tidal species; either diurnal or semidiurnal
4. Analyses of the geometry revealed a width convergent seaward reach and height convergent and sinuous landward reach in a microtidal diurnal system, suggesting the established fluvial‐marine geomorphic transition is broadly applicable to other systems. Analytical equations suggest tidal characteristics affect the transition length scales, making diurnal systems less convergent, longer, and subsequently more sensitive to river discharge effects (Equation 5)
5. In backwater reaches, river discharge events reduced height convergence and increased friction, making tidal currents attenuate faster and become more delayed with river discharge than tidal observations using a water level

We provide reduced complexity analytical solutions for estimating the shifting locations of the flood limit and river attenuation of tides (Equation (6a), (6b), (6c)) as well as tidal interactions with system geometry (Equation 5). Understanding these tide‐river‐geomorphic interactions are important now (e.g., sediment/nutrient transport, navigation, recreation) and in the future, as communities develop new infrastructure, insurance plans, and manage natural resources under the pressures of global warming, sea level rise, and altered river flows.

## Conflict of Interest

The authors declare no conflicts of interest relevant to this study.

## Supporting information

Supporting Information S1

## Data Availability

All data needed to recreate this analysis are publicly available through the NOAA (Tides & Currents, Mobile, Alabama DEM), the USGS (National Water Information System, 3DEP Lidar), Dykstra and Dzwonkowski (2020b), and Farr et al. (2007). Individual stations are further detailed in Table S1 of the Supporting Information S1.
