# Supplementary material for: The Role of River Discharge and Geometric Structure on Diurnal Tidal Dynamics, Alabama, USA
Source: J Geophys Res Oceans. 2022 Mar 28;127(3):e2021JC018007. doi: 10.1029/2021JC018007 (PMC9287036; doi:10.1029/2021JC018007)
Supplement: Supplementary file 1 — Supporting Information S1 [file JGRC-127-0-s001.docx]

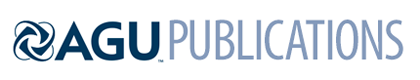


*Journal of Geophysical Research: Oceans*

Supporting Information for

**The role of river discharge and geometric structure on diurnal tidal dynamics, Alabama, USA**

S. L. Dykstra^1,2,3*^, B. Dzwonkowski^2,3^, and R. Torres^1^

^1^School of Earth, Ocean, and Environment, University of South Carolina, Columbia, SC 29208.

^2^Department of Marine Sciences, University of South Alabama, Dauphin Island Sea Lab, Dauphin Island, AL 36528.

^3^Dauphin Island Sea Lab, Dauphin Island, AL 36528.

**Contents of this file**

Figures S1 to S3

Table S1

Supporting Figures


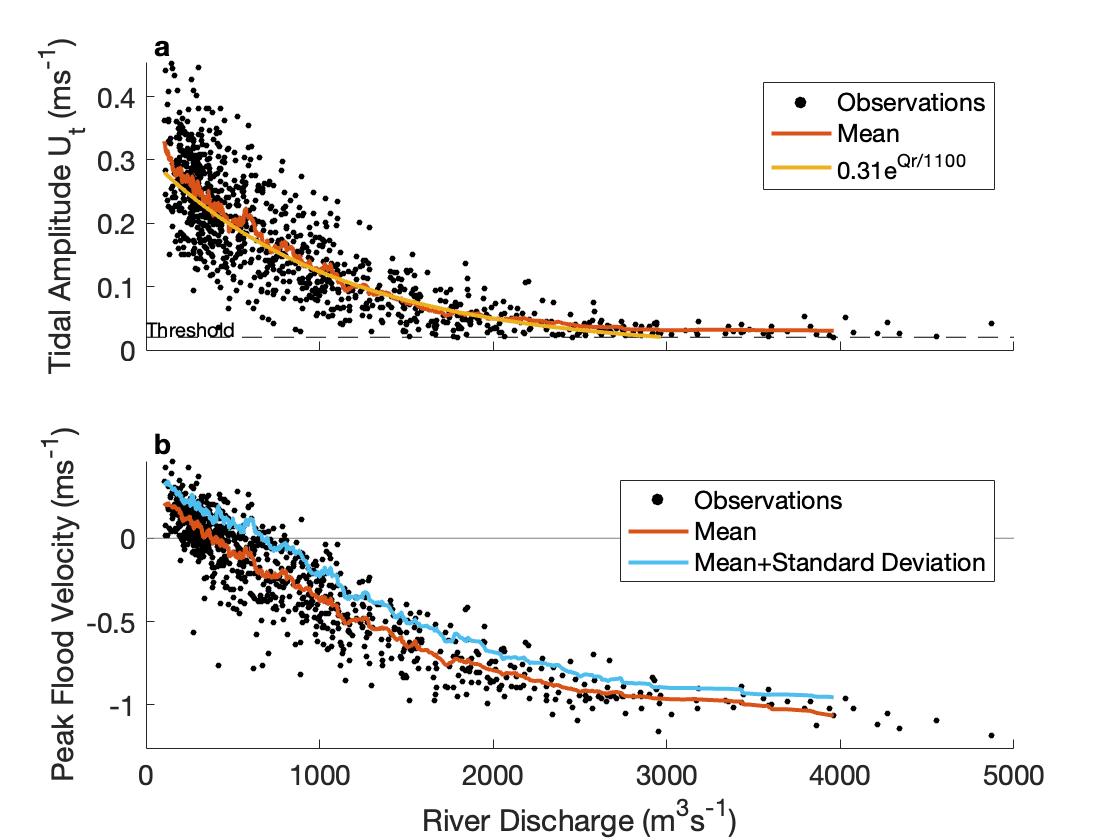


**Figure S1.** An example of river induced tidal attenuation at D100, showing changes in (a) tidal current amplitudes and (b) their corresponding velocities, capturing peak flood currents. (a) Amplitudes were found using the band-pass filtered velocity (3–40-hour Lanczos filter), capturing peaks above a 0.02ms^-1^ threshold with periods of 24.8±2 hours and removing outliers (n=1,024; black dots). Amplitude scatter along the y-axis captures spring-neap variability. To calculate the mean amplitude for a given river discharge, amplitudes were sorted by river discharge and averaged with a moving mean (red line). Using a box size of 31, the mean captured 33 degrees of freedom and closely matched a fitted decay curve (U_t_=0.31e^Qr/1100^; yellow line). (b) The time at which tidal amplitude peaked, in the unfiltered velocity, corresponds with peak flood velocity, from which a moving-mean and moving-standard deviation analysis was completed, also using a box size of 31. Using sinusoidal data (i.e., spring-neap cycle), the mean captures the 50^th^ percentile (red line) and the mean+standard deviation captures the 71^st^ percentile (blue line), which is representative of spring tides. As the peak flood velocity becomes smaller with river discharge, the moving mean+moving standard deviation line intercepts the x-axis, capturing the river discharge at which tides no longer reverse flow (i.e., flood limit). This analysis was applied to all velocity stations in figure 6b. (a) Because the tidal amplitude moving-mean ‘asymptotes’ near the threshold above 3,000 m^3^s^-1^, the amplitudes at higher river discharge are interpreted as noise.


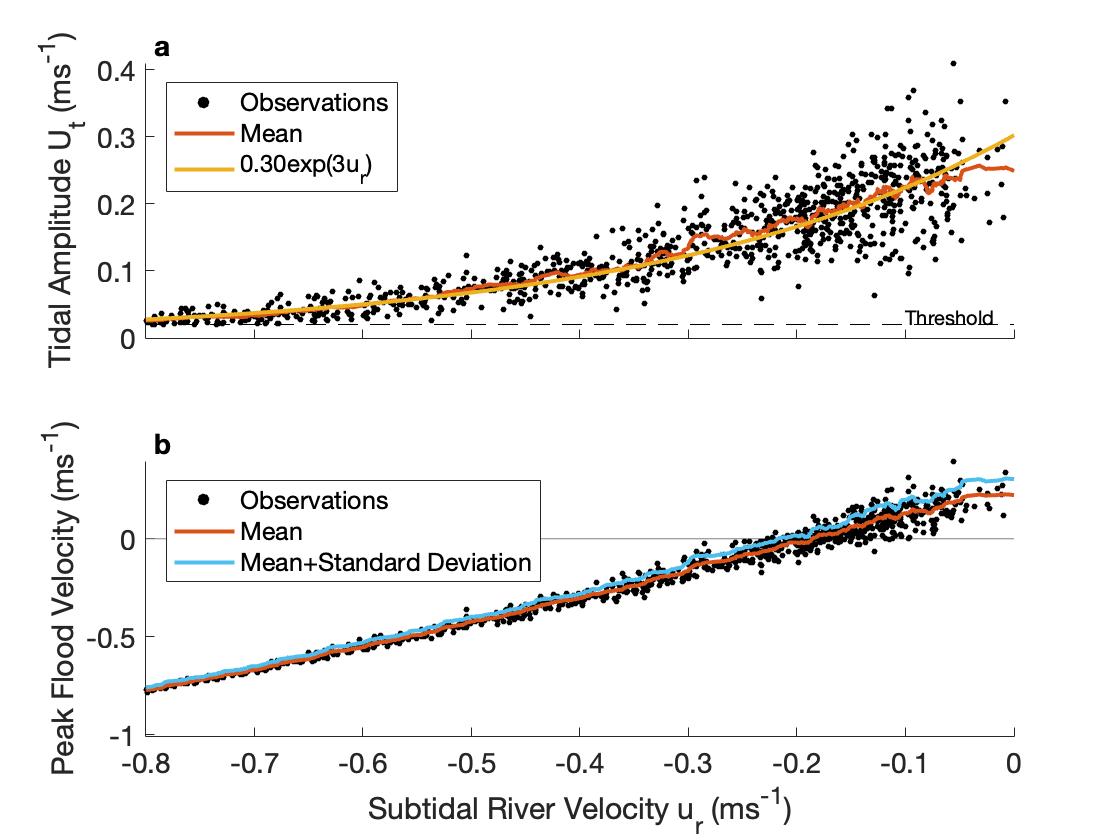


**Figure S2.** An example of river induced tidal attenuation at D100, same as Figure S1, but instead of river discharge on the x-axis, river velocity is shown (calculated using a 40-hour lowpass Lanczos filter). (a) Tidal amplitude decays exponentially with subtidal river velocity, approaching the threshold where u_r_/U_t_=Q_rf_~20, a valued used to approximate the location of the tidal limit (Figure 9). (b) The relationship between the subtidal river velocity and peak flood velocity, captured with the moving-mean (red line), is nearly linear and fits the function: flood velocity=1.30u_r_+0.24.


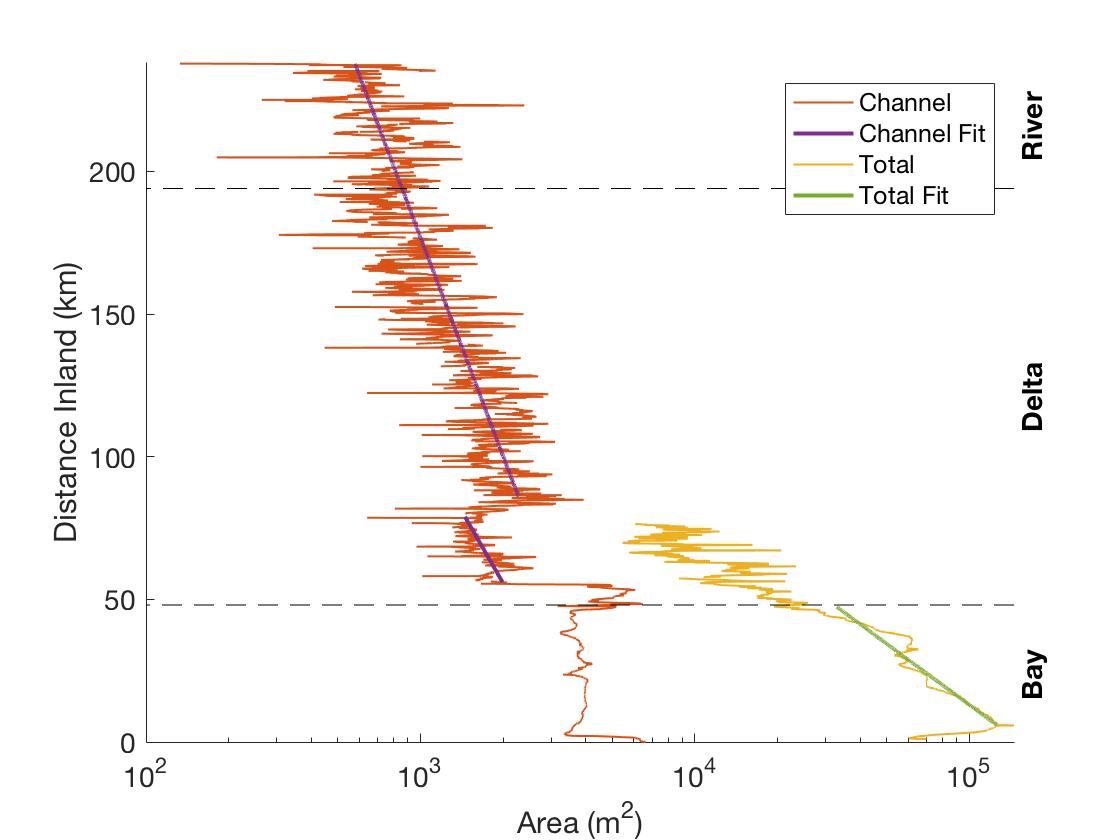


**Figure S3.** Area below sea level estimate of the channel, along the fluvial-marine transition (red, blue), and the total area, along the cardinal direction of 0 degrees north (yellow, purple). Log fits capturing convergence are completed following the delineated sections of figure 4 (blue, purple). Because the channel area fit for the Tombigbee River did not change by extending it to the geomorphic change point in the Mobile River (i.e. L_a_=111km), one line is shown. (Note: the channel estimate only captures the areas surveyed by the Army Corps of Engineers, missing some of the shoals. The total area landward of the bayhead (dotted line at 48km) does not match the distance inland along the sinuous longitudinal transect. Due to these limitations, this figure was not included as part of figure 4.)

**Supporting Tables**

**Table S1.** Station locations, length of records and data sources.
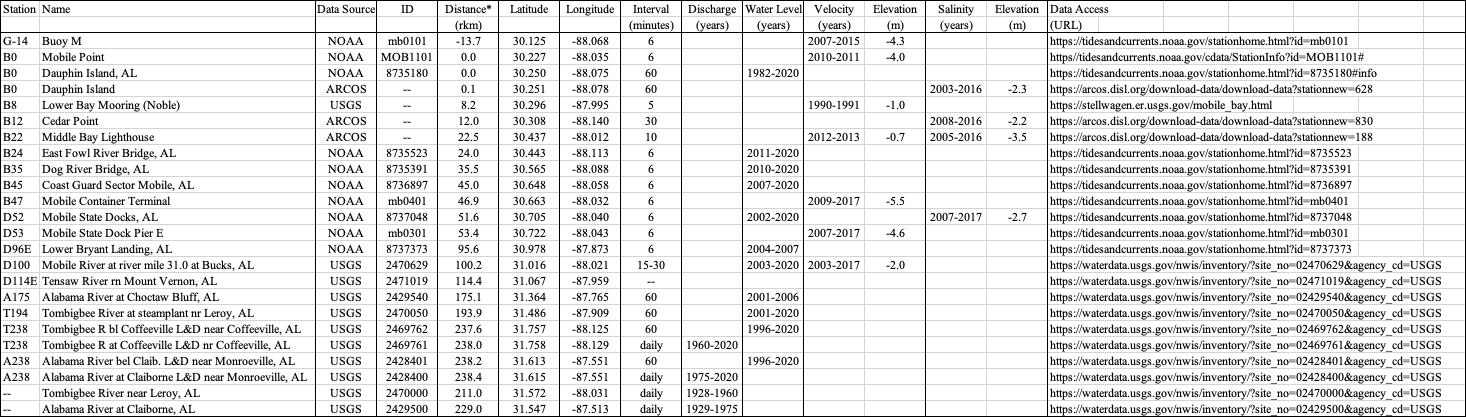


*distance inland from Main Pass

**Alterative data access is available through the National Center for Environmental Information (https://data.nodc.noaa.gov/cgi-bin/iso?id=gov.noaa.nodc:DISL_ARCOS;view=html) for B0 (0114998, 0141141, 0159582, 0172587), B12 (0117373, 0140929, 0159581, 0172811), and B22 (0117376, 0141138, 0159585, 0172809)
